# On the mechanism of photocatalytic reactions with eosin Y

**DOI:** 10.3762/bjoc.10.97

**Published:** 2014-04-30

**Authors:** Michal Majek, Fabiana Filace, Axel Jacobi von Wangelin

**Affiliations:** 1 Institute of Organic Chemistry, University of Regensburg, Universitaetsstr. 31, 93040 Regensburg, Germany; 2 Institute of Organic Chemistry, University of Alcalá, Alcalá de Henares, Madrid, Spain

**Keywords:** mechanism, organocatalysis, photocatalysis, photoredox catalysis, quantum yields, visible light

## Abstract

A combined spectroscopic, synthetic, and apparative study has allowed a more detailed mechanistic rationalization of several recently reported eosin Y-catalyzed aromatic substitutions at arenediazonium salts. The operation of rapid acid–base equilibria, direct photolysis pathways, and radical chain reactions has been discussed on the basis of pH, solvent polarity, lamp type, absorption properties, and quantum yields. Determination of the latter proved to be an especially valuable tool for the distinction between radical chain and photocatalytic reactions.

## Introduction

The ability of natural systems to harness solar energy for the genesis of matter has been fascinating mankind since time immemorial and has stimulated numerous reproduction attempts in the context of chemical synthesis over the last two centuries. The vast majority of photochemical reactions known until the 1980s exploited stoichiometric amounts of a photoactive molecular entity to drive a chemical transformation [1]. Only recently, a steadily growing number of homogeneous transition metal complexes which are redox-active and show absorption in the visible range of the solar spectrum have been demonstrated to catalyze light-driven organic reactions. The use of the pyridyl-based complexes [Ru(bpy)_3_]^2+^, [Ir(ppy)_3_] and [Ir(ppy_2_)(dtbbpy)]^+^ for the mediation of redox processes has certainly attracted the most interest, incipiently in photocatalytic reactions of activated organic electrophiles [2–8].

**Scheme 1:**
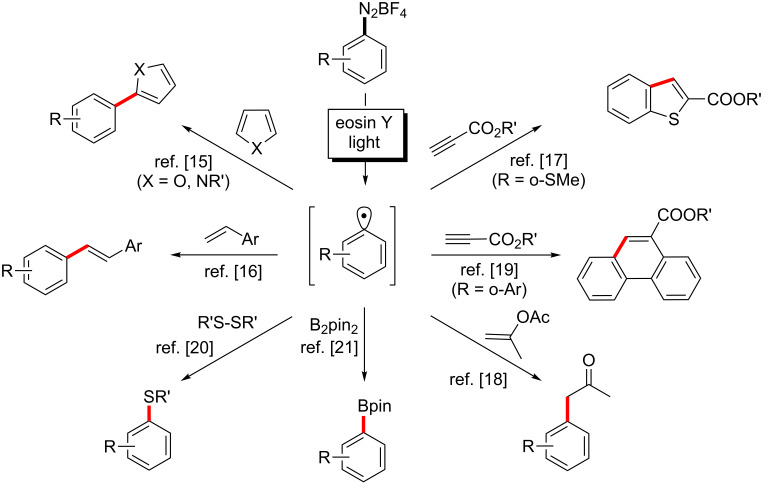
Oxidative quenching of eosin Y with arenediazonium salts and reactions of the resultant aryl radicals.

Despite the numerous examples of efficient catalytic photoredox transformations with organometallic dyes known to date, their high price, toxicity profile, and problematic recyclability might limit their more general use especially on larger scales. However, the recent pursuit of environmentally more benign photoactive catalysts has focused on much cheaper metal-free dyes. Several commercially available fluorescein and xanthene dyes have been successfully applied to photoredox reactions, including radical substitutions at α-amino, β-carbonyl, and aryl moieties [9–10]. Among them, eosin Y, the 2’,4’,5’,7’-tetrabromo derivative of fluorescein, has been most widely employed. The redox potential of the EY^+^/EY* pair of 1.1 V (vs. SCE) is experimentally not available as both of the compounds are short-lived intermediates. However, the redox potential can be obtained indirectly via analysis of the thermodynamic cycle involving the energy of the triplet state eosin Y* (T_1_) (derived from fluorescence measurements) and the energy of the radical cation eosin Y^+•^ (derived from cyclovoltammetric experiments, for more details see Supporting Information File 1). Much effort has been directed at the oxidative quenching of eosin Y* (T_1_) with suitable electrophiles in order to generate aryl radicals by a light-driven single-electron transfer (SET) process (i.e., one-electron reduction of Ar–X, see Scheme 1). Due to their easy reducibility, arenediazonium salts are especially attractive precursors which constitute versatile alternatives to haloarene-based strategies. They are readily available by diazotization of anilines, no toxic metals are required; the bond cleavage generates gaseous N_2_ which escapes the reaction mixture. Photoredox reactions with arenediazonium salts are often more selective than traditional methods such as copper(II)-mediated Meerwein arylations [11] or protocols employing stoichiometric iron(II) or titanium(III) reductants in aqueous media [12–14]. This renaissance of arene diazonium chemistry has recently led to various applications of eosin Y to visible light-driven syntheses of biaryls [15], stilbenes [16], benzothiophenes [17], α-phenylketones [18], phenanthrenes [19], arylsulfides [20] and arylboron pinacolates [21] (Scheme 1).

**Scheme 2:**
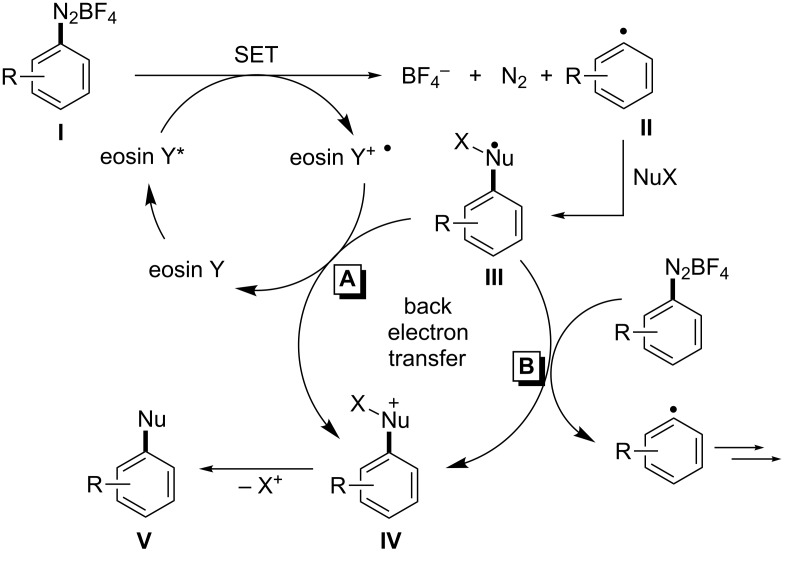
Proposed general reaction mechanism of eosin Y-catalyzed substitutions with arene diazonium salts.

Numerous mechanistic studies have been performed at reactions with organometallic photocatalysts [3–8], whereas much less attention has been directed at eosin Y-catalyzed reactions. The reductive quenching pathway of eosin Y, which operates in the photooxidation of isoquinolines [9], has been studied in a single report [22]. To the best of our knowledge, related data have not been collected for the much more widely used oxidative quenching. Most literature protocols were interpreted in analogy to the related [Ru(bpy)_3_Cl_2_]-catalyzed reactions and similar mechanisms were proposed (Scheme 2) [15–21]. These generally commence with the SET between excited eosin Y and the arenediazonium salt **I** to give aryl radical **II**. Nucleophilic attack onto reactive **II** generates the more stable complex **III** which is prone to back-electron transfer upon oxidative formation of the cationic species **IV**. Terminal elimination of X^+^ (mostly a proton) gives the substitution product **V**. Two general pathways of back-electron transfer can be followed: Path A involves one-electron reduction of the radical cation state of the catalyst. Radical chain propagation (B) can occur when the SET occurs with another molecule of the starting material **I**. Some attempts have been made to differentiate between radical chain and photocatalysis mechanisms by monitoring the reaction progress after the light sources have been switched off [20]. Clearly, such experiments neglect the existence of short radical chains and thus provide no conclusive evidence of the mechanism.

In general, the thermodynamic feasibility of a redox process is determined by the difference of redox potentials of the two half reactions. The redox potential of most arenediazonium salts (redox pair **I**/**II**, Scheme 2) is close to 0 V vs. SCE [23–24]. The redox potentials of the short-lived adducts **III**/**IV** are unknown and experimentally not (easily) available. However, it is likely that their potential is greater than 0 V in many cases which makes the radical species **III** a sufficiently strong reductant for arenediazonium salts of type **I**. After all, the unambiguous determination of the underlying reaction mechanisms is not possible without further spectroscopic, kinetic, and theoretical experiments. The studied systems are mostly too complex for transient absorption spectroscopy. We have so far failed to obtain any insight from photo-CIDNP NMR experiments. Therefore, we have decided to evaluate the efficiency of the radiation events of several recent literature protocols by recording the quantum yields Φ [15–17,19–21]. Furthermore, we have learned along the way that the reactions appear to be strongly dependent on the pH of the solution and the type of lamp used for irradiation. Here, we present a detailed study on the direct consequences of these three major factors for the outcome and mechanism of several recently reported eosin Y-catalyzed aromatic substitution protocols starting from arenediazonium salts [15–21].

## Results and Discussion

### Effect of pH on the efficiency of eosin Y-mediated photocatalysis

**Figure 1:**
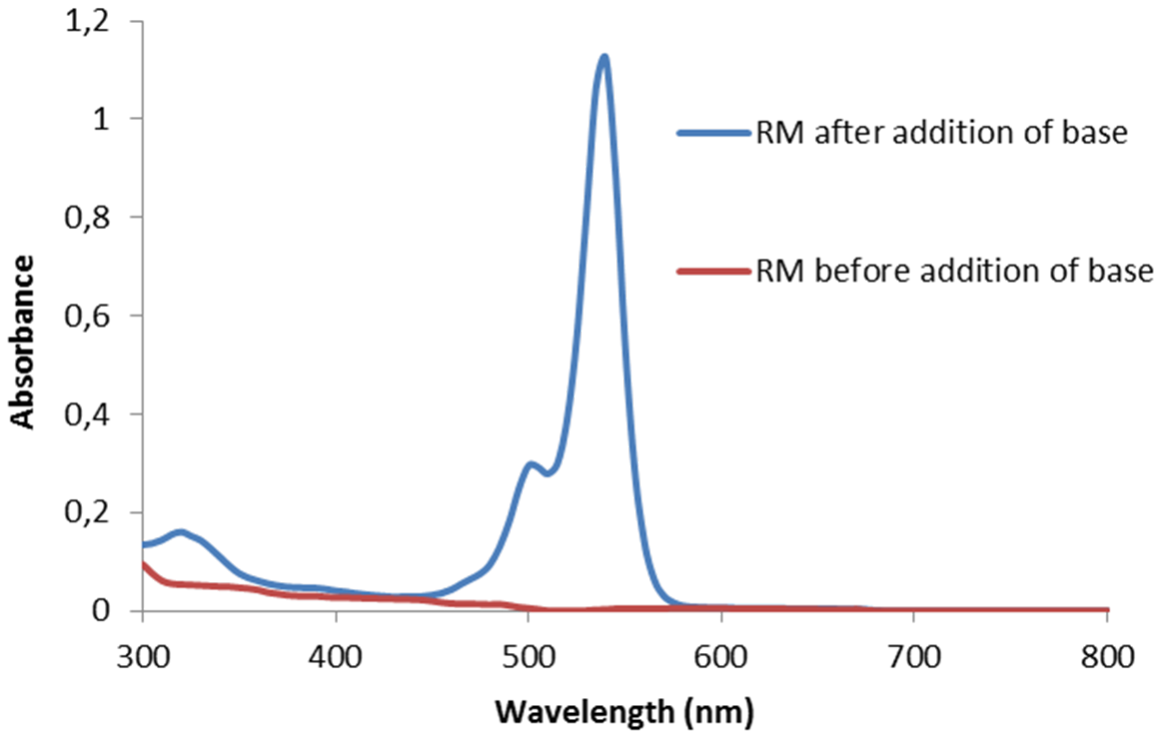
UV–vis spectra of the photoborylation reaction mixture (RM).

**Figure 2:**
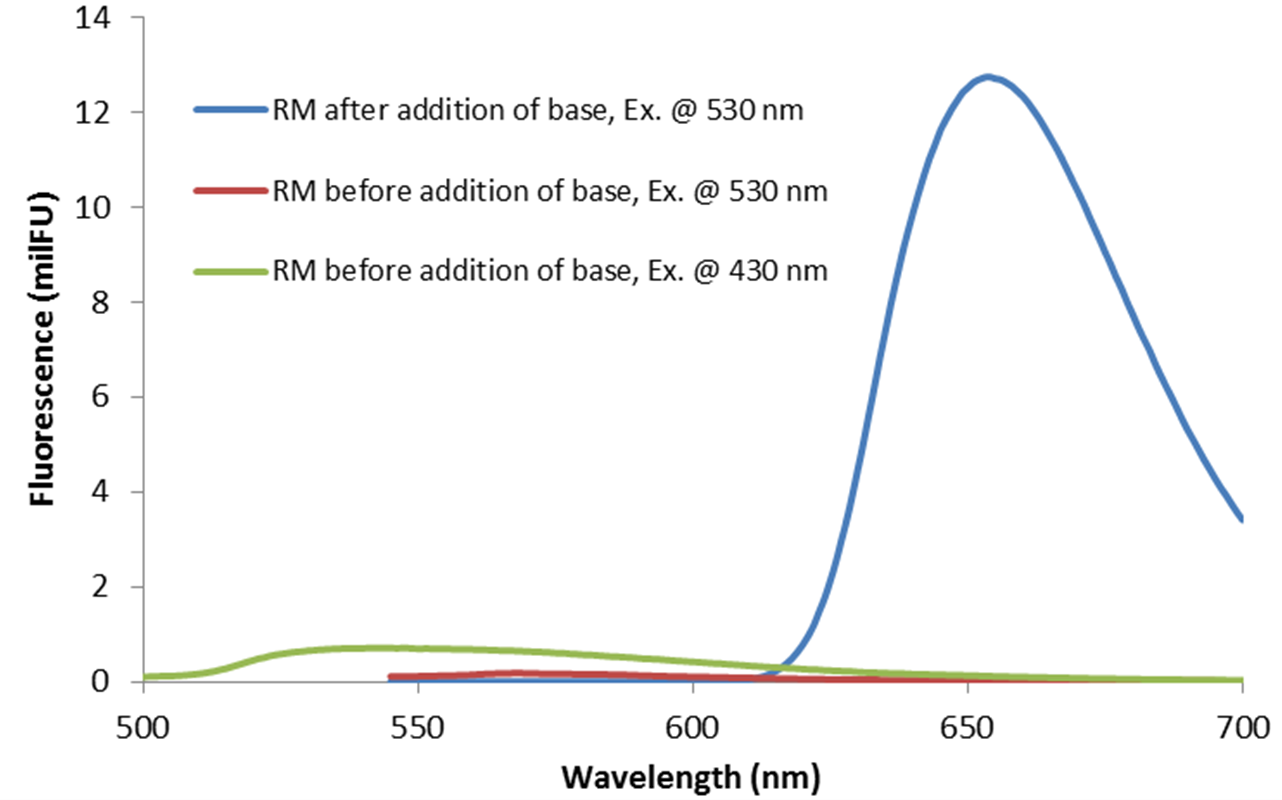
Fluorescence spectra of the photoborylation reaction mixture (RM). Ex. = excitation wavelength.

For better comparison, we have employed identical reagent concentrations and reaction conditions (solvents) as reported in the original papers [15–21]. Dry DMSO was mostly used as solvent, with the exception of the phenanthrene [19] and arylboron pinacolate [21] syntheses which were run in acetonitrile. Much to our surprise, the solutions of eosin Y and arenediazonium salts in acetonitrile were yellow (instead of the usual red colour; see Figure 1, conditions as in [21]) and did not exhibit intense fluorescence (Figure 2, conditions as in [21]). We have suspected this to be a consequence of facile acid–base reactions of the catalyst eosin Y. Indeed, immediate colour change was effected by addition of base (tetra-*n*-butylammonium hydroxide, TBAOH), and strong fluorescence occurred in the green spectrum. This dramatic pH effect on the spectroscopic properties of eosin Y solutions prompted us to further investigate this behaviour.

**Scheme 3:**
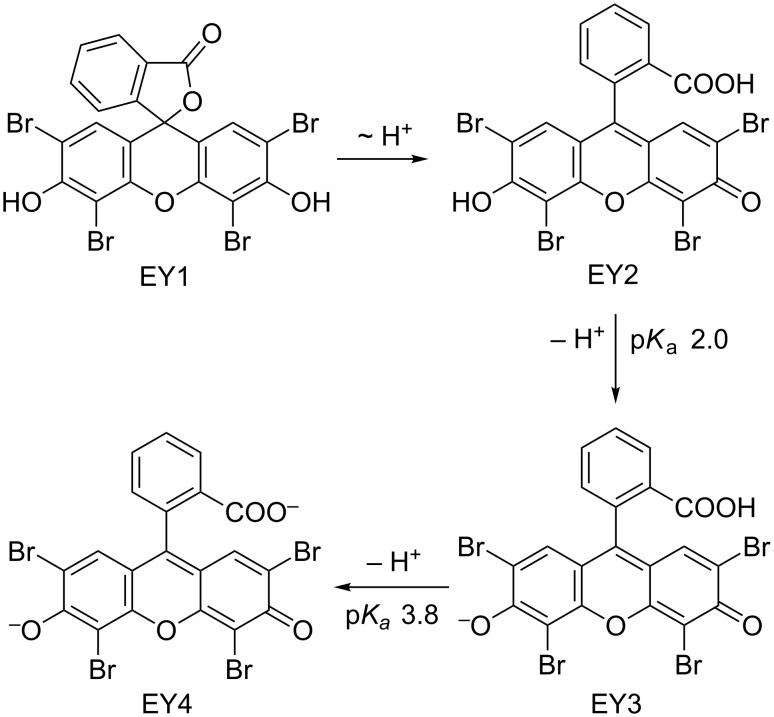
Acid–base behaviour of eosin Y.

The organic dye eosin Y can exist in four different structures in solution: the spirocyclic form EY1, the neutral EY2, the monoanionic EY3, and the dianionic form EY4 (Scheme 3). Eosin Y contains two relatively acidic protons (p*K*_a_ 2.0, 3.8 in water) [25] which can be easily abstracted to give dianionic EY4. Lack of clarity exists in many publications on photoredox catalysis with regard to the nature of the employed dye. The authors either report the use of “eosin Y, spirit soluble” – which can be EY1 or EY2 according to the Sigma–Aldrich catalogue [26], or claim the use of the free acid EY2. The presence of stoichiometric amounts of bases, e.g., in eosin Y-catalyzed photo-oxidative transformations with amines [9], results in the quantitative generation of the dianionic form EY4 under the reaction conditions. On the other hand, the SET-generation of aryl radicals from arenediazonium salts by photocatalysis proceeds in the absence of base. The non-aqueous conditions should not provide a significant buffering capacity. Here, the presence of only minute amount of impurities in the solvents or starting materials is sufficient to push the acid–base equilibria of the catalytic amounts of eosin Y in either direction.

The spirocyclic form EY1 contains an interrupted conjugation of the fluorone ring system and thus would be photocatalytically inactive under visible light irradiation. The neutral form EY2 exhibits only weak fluorescence when irradiated with visible light (Figure 2). Studies on related alkylated eosin derivatives suggest that this fluorescent state is very short lived and is therefore also not appropriate for photoredox catalysis [27]. The charged forms EY3 and EY4 are catalytically active, which in turn also means that solutions containing commercial “eosin Y, spirit soluble” would per se not trigger efficient photoredox catalysis. In such cases, catalytic activity as observed in recent publications by König et al. is dependent on the operation of acid–base equilibration (e.g., in DMSO solution) so that the employed eosin Y is converted in situ to the active species EY3 or EY4 [15–18]. Efforts to reproduce the photoredox synthesis of arylboron pinacolates in acetonitrile have so far failed in our hands when irradiating at 535 nm [21]. No product formation was observed, and the UV–vis spectra showed no appreciable absorption at this wavelength. However, the addition of minor quantities of base (TBAOH) to the reaction mixture resulted in strong absorption at 535 nm and good photocatalytic activity under irradiation. The substitution of acetonitrile with DMSO in the same reaction gave strong absorption at 535 nm and allowed the photocatalytic synthesis of the desired arylborinic ester in good yields in the absence of extra base. This discrepancy is most likely associated with the different properties of DMSO and acetonitrile as bases. DMSO is a stronger base than acetonitrile which enhances the acidity of most Brønsted acids in DMSO [28]. These observations lead to the conclusion that photoredox reactions catalyzed by eosin Y (or similar organic dyes) cannot be discussed without strict specification of the employed form of the dye and the reaction conditions. Conclusive mechanistic proposals of visible-light-driven reactions with these dyes also need to address the operation of acid–base equilibria and the pH dependence of absorption properties.

### Effect of the light source on the eosin Y-mediated photocatalysis

Another reaction parameter which lacks clarity and consistency among the literature reports is the source of irradiation. Several groups including ourselves have used commercial narrow-band LEDs with a maximum intensity at 535 nm (green light) [15–18,20]. Other reactions were irradiated with white light from broad-band compact fluorescent lamps (CFL) [19,21]. The determination of quantum yields requires the use of narrow-band light sources due to the variation of the optical density of the samples with the wavelength. Therefore, we have studied the impact of different irradiation types on the course of the photocatalytic reaction. Although the type of CFLs was not specified in the literature [19,21], the majority of commercial CFLs cover similar spectral ranges with the individual UV edge being significantly below 400 nm and with substantial radiation power in the region of 400–500 nm. A similar wavelength distribution is seen in the spectrum of commercial white-colored LEDs (see Supporting Information File 1 for spectrum). The activation energy of thermal heterolysis of arenediazonium ions is approx. 115 kJ/mol (1.19 eV) [29]. The energy of a photon at the edge of the visible spectrum (400 nm) is 3.1 eV so that such photons carry sufficient energy to heterolyze diazonium ions to give highly electrophilic aryl cations. Moreover, arenediazonium salts are known to form weak charge-transfer complexes with solvent molecules whose absorption tails into the visible range [30].

**Figure 3:**
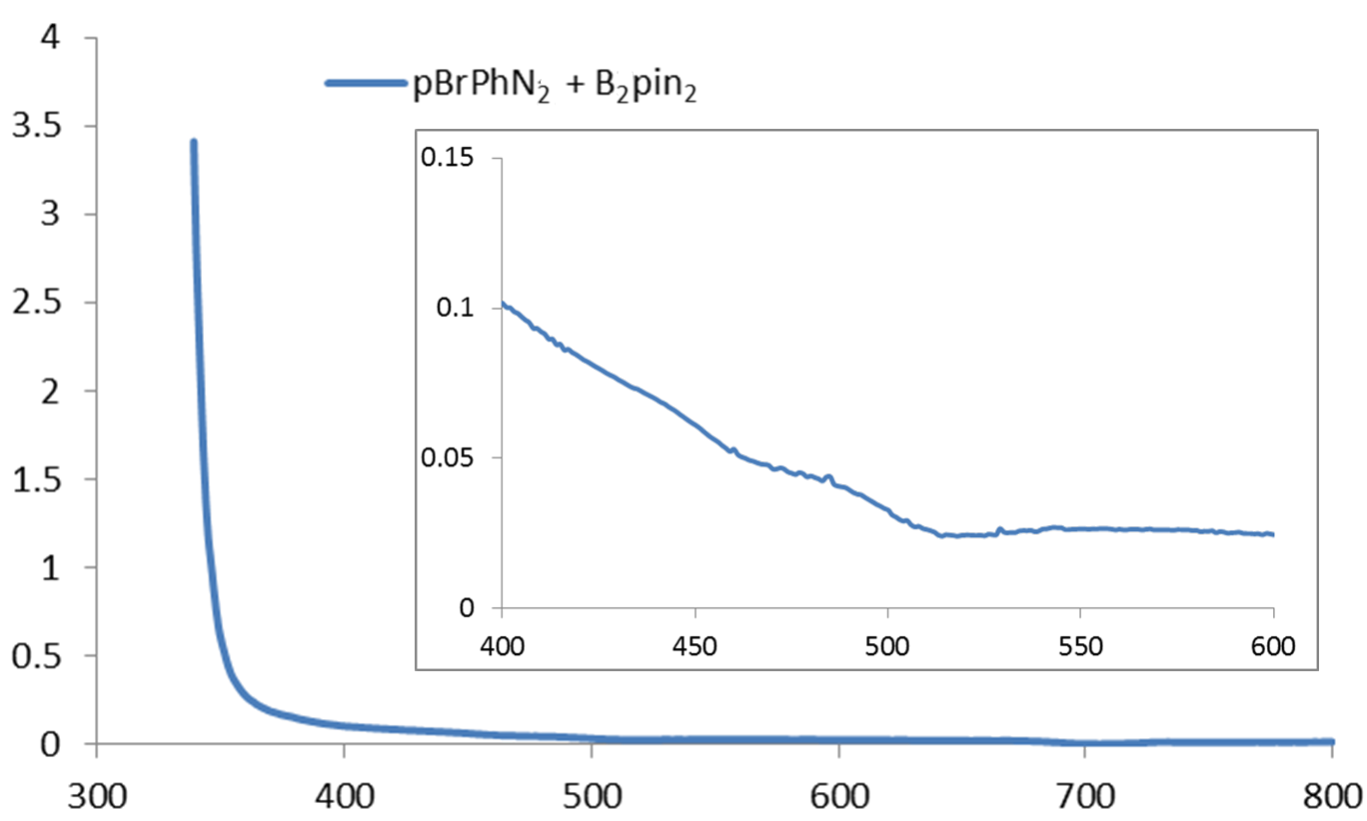
UV–vis spectrum of *p*-bromobenzenediazonium tetrafluoroborate (pBrPhN_2_) and bispinacolato diboron (B_2_pin_2_) in acetonitrile.

**Scheme 4:**
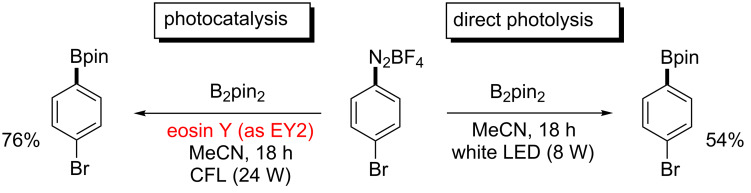
Eosin Y-catalyzed and dye-free photolytic borylation.

These observations support the notion that the use of broad-band visible irradiation indeed can have a profound effect on the outcome of a photocatalytic reaction. We have therefore examined if direct absorption of the arenediazonium ions can trigger a productive pathway under irradiation with broad-band light sources even in the absence of the photocatalyst. As representative examples we chose the recently reported syntheses of arylborinic esters [21] and phenanthrenes [19]. The absorption spectrum of a mixture of *para*-bromobenzenediazonium tetrafluoroborate (*p*-BrC_6_H_4_N_2_BF_4_) and bispinacolato diboron (B_2_pin_2_) in acetonitrile shows a significant shoulder of a UV-absorption band tailing into the visible part of the spectrum (Figure 3) [30]. At the UV–vis edge (400 nm), the absorbance of the system is still more than 0.1 which translates into 21% of all light being absorbed at this wavelength. When we performed the borylation reaction according to the literature report [21] but without the addition of the photocatalyst eosin Y, 54% yield of the borylation product were obtained by direct photolysis (Scheme 4). These observations are in full accord with a report of direct reaction of thermally generated aryl cations (from arenediazonium salts) with bispinacolato diboron to give the corresponding arylborinic esters [31]. It is thus very likely that direct light-triggered heterolysis of the starting material accounts for substantial amounts of product formation under conditions which were believed to proceed through photocatalytic SET.

**Scheme 5:**
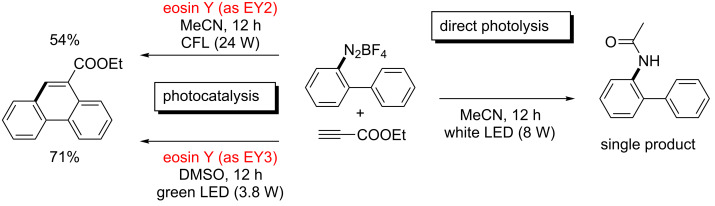
Eosin Y-catalyzed and dye-free reactions with ethyl propiolate.

**Figure 4:**
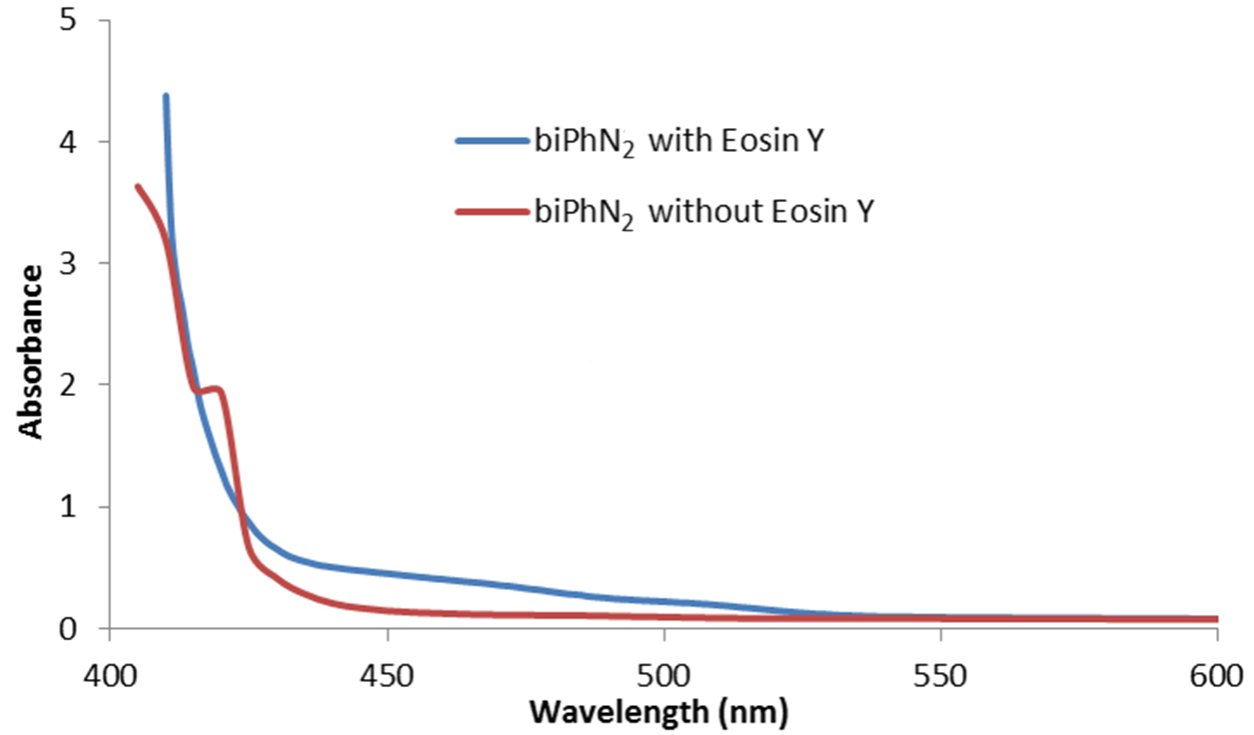
UV–vis spectra of *ortho*-biphenyldiazonium tetrafluoroborate (biPhN_2_) in acetonitrile.

A similar behaviour was found in our study of the phenanthrene synthesis [19]. A solution of *o*-biphenyldiazonium tetrafluoroborate in acetonitrile showed strong absorption between 400–500 nm with all light at the UV–vis edge at 400 nm being completely absorbed. This again indicates that direct photocleavage of the C–N bond might be operating. The cyclization reaction with ethyl propiolate according to the literature protocol [19] but in the absence of eosin Y did not afford any phenanthrene product (Scheme 5). Instead, a Ritter-type reaction proceeded to give the corresponding acetanilide after aqueous work-up which is consistent with the original report by Deronzier from 1984 [32]. The significant overlap of the absorption spectra of the arenediazonium salt recorded before and after the addition of eosin Y (Figure 4) suggests that direct photolysis of the C–N bond could account for the erosion of product yield in the catalytic process due to competitive heterolysis of the substrate and subsequent ionic Ritter reaction. This notion was supported by our experiments performed in DMSO where a higher portion of the photocatalyst resides in the active EY3 and EY4 states. Following an otherwise identical protocol, irradiation at 535 nm resulted in the formation of the phenanthrene in 71% yield (cf. literature yield of 53%)[19].

### Quantum yields of eosin Y-catalyzed reactions

The determination of quantum yields of light-driven reactions provides valuable insight into the efficiency of the radiative processes and thus can be used for the mechanistic understanding of such processes. The magnitude of quantum yields Φ also describes how much energy is wasted into thermal dissipation in such systems, which is an especially critical parameter for the evaluation of sustainability of a photocatalytic process. The quantum yield is defined as the efficiency of a photochemical reaction in the studied system:

Φ = (rate of substrate conversion)/(absorbed photon flux)

Theoretically, if a simple photocatalytic process is considered, the quantum yield would be in the range 0 < Φ ≤ 1. It approaches unity as the efficiency of the photocatalytic step increases. In reality, quantum yields can exceed unity in cases where the products of the photocatalytic reaction induce (radical) chain reactions. Therefore, the determination of quantum yields Φ provides a meaningful answer to mechanistic ambiguities. For the eosin Y-catalyzed reactions with arenediazonium salts, conclusive answers to the distinction between photocatalytic and radical chain mechanisms can be derived directly from Φ.

A quantification of the photon flux is rather problematic. Recently, devices became available which use solar cells for the direct measurement of photon fluxes [33]. However, chemical actinometry constitutes a prevalent indirect method of photon flux measurement [34]. Several effective chemical actinometers are known for UV spectral studies whereas similar experiments in the visible range are much more limited by the availability of chemical actinometers. Even more challenging are photon flux determinations above 500 nm which marks the spectral cut-off of the commonly used Hatchard–Parker ferrioxalate. None of the chemical actinometers that operate in this region are commercially available. We therefore decided to prepare potassium Reineckate, a robust actinometer for the >500 nm region, according to a literature method [34–35].

**Scheme 6:**
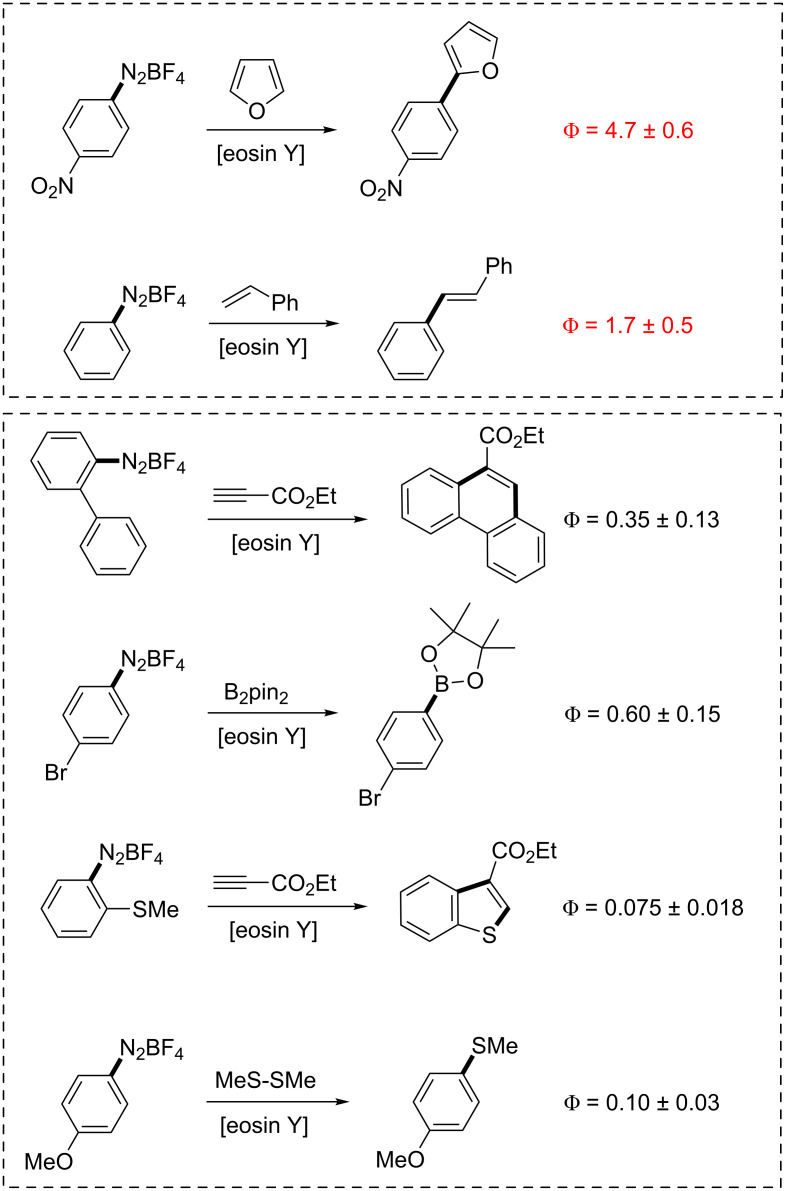
Quantum yield determinations of selected visible-light-driven aromatic substitutions.

Quantum yields Φ were measured for all aforementioned visible-light-driven reactions in the same solvent, DMSO. Irradiation was performed with a green LED (3.8 W) at 535 nm. All other reaction conditions were adopted from the individual literature reports [15–21]. For more details on the actinometry experiments and quantum yield determinations, see Supporting Information File 1. The observed quantum yields Φ of the studied reactions varied by almost two orders of magnitude, between 4.7 and 0.075. This already indicates the operation of different mechanisms in these aromatic substitution reactions with arene diazonium salts. The redox potentials of most substituted arene diazonium salts cluster with very little deviation around 0.0 V vs. SCE (±0.2 V) [23–24] so that the observed differences in Φ can be largely attributed to different mechanistic pathways. Our experiments (Scheme 6) afforded quantum yields of Φ > 1 for the heterobiaryl coupling [15] and the Heck-type olefination with styrene [16], respectively. This indicates that in addition to a photocatalytic path (Scheme 2, A) radical-chain propagation is operating under the reaction conditions (Scheme 2, B). This contrasts with the other reactions where the quantum yields range between 0.6 and 0.075. In the original paper from Deronzier [32], where similar reactions under catalysis of Ru(bpy)_3_^2+^ were studied, Φ values of 0.46–0.78 were reported. Our actinometric experiments of the eosin Y-catalyzed phenanthrene synthesis [19] and photoborylation [21] gave similar quantum yields of 0.35 and 0.60, respectively. This suggests that the photocatalytic pathway is indeed populated (Scheme 2, A). Finally, the benzothiophene synthesis [17] and photothiolation [19] exhibited relatively low quantum yields which could be a consequence of non-productive processes that are responsible for the significant loss of energy. The presence of electron-rich alkylthiolate moieties in both systems could in principle effect reversible redox processes with the catalyst eosin Y which might account for the erosion of Φ.

## Conclusion

In summary, we have investigated the impact of several reaction parameters on the outcome and mechanism of photocatalytic aromatic substitution reactions of arenediazonium salts in the presence of eosin Y. However, the significance of these data certainly extends to other light-driven reactions that lie beyond the focus of this study. Eosin Y (and many other organic photocatalysts) undergo rapid acid–base equilibria which significantly alter the photophysical properties. It is therefore of pivotal importance to ascertain the actual nature of the employed dye under the reaction conditions. Experimental details should always be given that specify the employed dye, the presence of acids and bases as well as the purity of the reagents, solvents, and additives. The use of broad-spectrum lamps in photocatalytic reactions with arenediazonium salts is strongly discouraged as they promote heterolytic C–N bond cleavage toward highly reactive aryl cation species. The standard reaction conditions of many literature reports involve concentrations which are orders of magnitude higher than those suitable for absorption spectroscopy studies. This means that even very inefficient transitions (at tailings of absorption maxima) can indeed trigger productive processes and therefore need to be addressed in mechanistic rationalizations. Quantum yield determinations with potassium Reineckate have now allowed the distinction between photocatalytic and radical-chain mechanisms. However, the operation of the prevalent pathway is likely dictated by the stability of the relevant catalytic intermediates.

## Supporting Information

File 1 Experimental and analytical data.
