# Supplementary material for: On the mechanism of photocatalytic reactions with eosin Y
Source: Beilstein J Org Chem. 2014 Apr 30;10:981–9. doi: 10.3762/bjoc.10.97 (PMC4077370; doi:10.3762/bjoc.10.97)

Supporting Information

for

On the mechanism of photocatalytic reactions with eosin Y

Michal Majek*[1], Fabiana Filace[2] and Axel Jacobi von Wangelin*[1]

Address: [1]Institute of Organic Chemistry, University of Regensburg, Universitaetsstr. 31, 93040 Regensburg, Germany and [2]Institute of Organic Chemistry, University of Alcalá, Alcalá de Henares, Madrid, Spain

Email: Axel Jacobi von Wangelin - axel.jacobi@ur.de; Michal Majek - michal.majek@ur.de

*Corresponding author

Experimental and analytical data

General methods

Commercial chemicals were used as obtained from Sigma-Aldrich or Fisher. Solvents were used without further purification. For most reactions, DMSO dried over molecular sieves (certified <0.005% water content, Sigma-Aldrich) was used. TLC was performed on commercial silica gel coated aluminium plates (DC60 F254, Merck). Visualization was done with UV light. Product yields were determined by quantitative GC-FID. Authentic product samples were synthesized according to the cited literature reports [1-7]. Purity and structure were confirmed based on ^{1}H NMR, ^{13}C NMR, GCMS and ^{19}F NMR (where appropriate). NMR spectral data were collected on a Bruker Avance 400 (400 MHz for ^{1}H; 100 MHz for ^{13}C) spectrometer at 25 °C. Chemical shifts are reported in δ/ppm, and coupling constants *J* are given in Hertz. Solvent residual peaks were used as internal reference for all NMR measurements. Abbreviations: s – singlet, d – doublet, t – triplet, q – quartet, m – multiplet, dd – doublet of doublet.

Synthesis of the chemical actinometer

Potassium Reineckate: Attempts to reproduce the one-step synthesis by Szychlinski et al. [8] were not successful (operation was discontinued after formation of copious almond-smelling gases was observed during synthesis). Therefore, the synthesis was performed via a two-step procedure:

A: Synthesis of ammonium Reineckate by a modified protocol according to Dakin:[9] Ammonium thiocyanate (100 g, 1.3 mol) was heated in a beaker in an oil bath (oil bath temperature 150 °C) until a homogenous molten mass was formed. Then, a finely powdered mixture of ammonium dichromate (20 g, 68 mmol), and ammonium thiocyanate (20 g, 0.26 mol) was added in small portions during which the mixture

was rapidly stirred with a glass rod. Vigorous evolution of gas was observed when approx. 1/10 of the dichromate–thiocyanate mixture has been added. After the completion of addition (~20 min), the reaction was agitated with a glass rod for another 30 min, after which the heating was stopped. The mixture was further vigorously stirred and solidified on cooling. (Stirring prevents the formation of an indivisible solid!) The cooled solids were finely powdered in a mortar and added to an ice-water mixture (80 mL). The resultant mixture was stirred for 10 min, then the insoluble portion was filtered off. The collected precipitates were dissolved in hot water (60 °C, 200 mL), and the resultant mixture quickly filtered to remove any residual precipitate. The filtrate was placed in a refrigerator (6 °C) for 12 h. The formed crystals were filtered and washed on the filter with copious amounts of cold water until the filtrate was free of SCN^{-} ions (checked by reaction with Fe^{III}). After drying on air, ammonium Reineckate monohydrate (ammonium tetrathiocyanato-diamminechromate(III) monohydrate) was obtained as deep violet crystals (34.7 g, 72% yield). The dried product obtained by this method can be stored at room temperature under ambient light conditions for several months without decomposition. Attempts to further purify the crystals by crystallization from water–ethanol mixtures were unsuccessful but formed toxic HCN gas.

B: Synthesis of potassium Reineckate:

All operations were carried under red light (11 W, from Osram, bought in the Slovak Republic) in a dark room with exclusion of other forms of irradiation. An aqueous solution of KNO_3 (50 mL, saturated at ambient temperature) was heated up to 50 °C. Then, ammonium Reineckate monohydrate (2.0 g, 5.6 mmol) was added in one portion and the mixture was stirred for 5 min. The resultant solution was cooled to 6 °C in a refrigerator upon which crystals formed. The crystals were filtered and

washed with a minimal amount of cold water. After drying on air, potassium Reineckate (potassium tetrathiocyanatodiamminechromate(III) monohydrate) was obtained as deep violet crystals (1.7 g, 84% yield). Potassium Reineckate readily decomposes upon exposure to ambient light, even in the solid phase.

Chemical actinometry and quantum yield measurements:

Actinometry measurements were performed using a method according to Wegner and Adamson [10,11] with potassium Reineckate. Quantum-yield measurements were performed in the regime of total absorption of the incoming light by the dye. Irradiation was performed with a green high-power LED (Luxeon Rebel, Canada, P = 3.8 W, λ_{max} = 535 nm). The photon flux interacting with the sample was determined to be $1.5 \cdot 10^8$ photons per second (corresponding to irradiance of 0.55 μW/m^2 at the point of intercept of radiation with cuvette wall) which did not drift over the time of the measurements as confirmed by repetitive experiments. The individual reaction times were chosen so that conversion was kept below 10% as required by the used protocol [10,11]. The substrate conversions were determined by quantitative GC-FID analysis with *n*-pentadecane as internal standard. All quantum yields were determined as an average of at least three subsequent measurements.

Redox potential of eosin Y:

Knowledge of the exact redox potential of the pair eosin $Y^{+\cdot}$/eosin Y* (T_1) is central to the discussion of reaction mechanisms involving redox steps. This value is experimentally not available as both of the compounds are short-lived intermediates. However, the redox potential can be obtained indirectly via analysis of the following thermodynamic cycle:

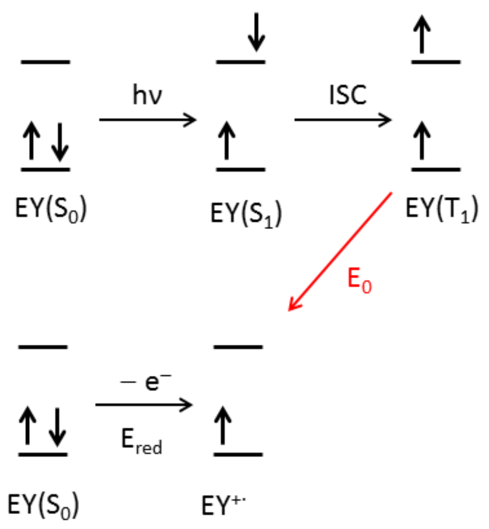

The energy of the triplet state eosin Y* (T_1) is derived from fluorescence measurements [12]: ΔE(triplet) = E(eosin Y(T_1)) − E(eosin Y(S_0)) = 1.89 eV.

The energy of the radical cation eosin $Y^{+\cdot}$ is derived from cyclovoltammetric experiments [13]: ΔE_{red} = E(Eosin $Y^{+\cdot}$) − E(eosin Y(S_0)) = 0.78 eV.

The combination of both values according to the aforementioned thermodynamic cycle allows the calculation of E_0:

ΔE_0 = ΔE(triplet) − ΔE_{red} so that $\boldsymbol{E_0}$ **= 1.11 eV**, therefore the redox potential of the eosin $Y^{+\cdot}$/eosin Y* (T_1) couple is −1.11 V**.**

Emission spectrum of the white LED light source

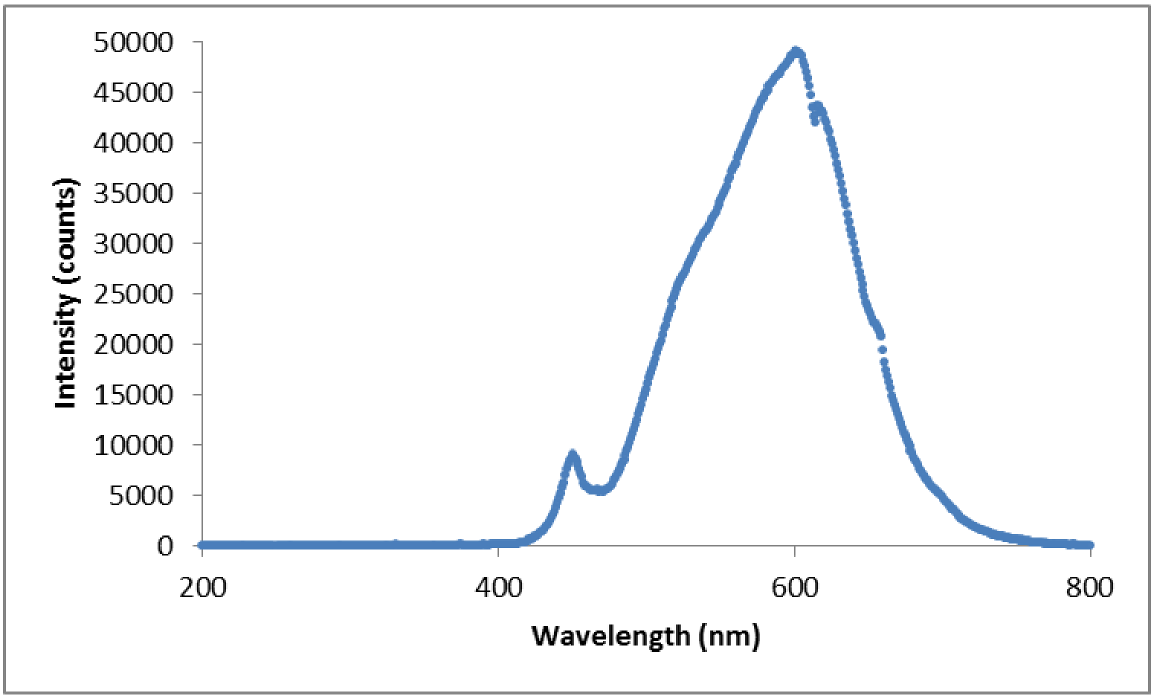

A white LED lamp with the upper spectrum was used for experiments that involved irradiation with white light.

Spectral data of the synthesized compounds

All products used as calibration standards for GC-FID quantification were synthesized by literature methods [1-7]. Stilbene was used as internal reference (Sigma–Aldrich).

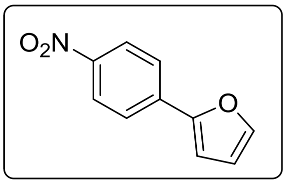

2-(4-Nitrophenyl)furan: ^{1}H NMR (400 MHz, $CDCl_3$, ppm) δ 8.25 (d, *J* = 9.0 Hz, 2H), 7.79 (d, *J* = 9.0 Hz, 2H), 7.58 (dd, *J* = 1.7 Hz, *J* = 0.5 Hz, 1H), 6.88 (dd, *J* = 3.5 Hz, *J* = 0.5 Hz, 1H), 6.56 (dd, *J* = 3.5 Hz, *J* = 1.7 Hz, 1H).^{13}C NMR (100 MHz, $CDCl_3$, ppm) δ 151.8 (C), 146.5 (C), 144.2 (CH), 136.5 (C), 124.4 (CH), 124.0 (CH), 112.5 (CH), 109.0 (CH). GC-MS (EI) *m/z* (relative intensity): 189 (100) [M^+], 159 (68), 131 (36), 115 (68), 89 (23).

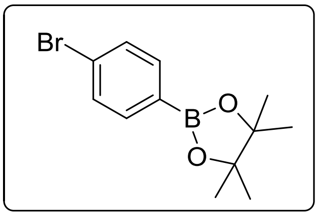

2-(4-Bromophenyl)-4,4,5,5-tetramethyl-1,3,2-dioxa-borolane: ^{1}H NMR (400 MHz, $CDCl_3$, ppm) δ 7.66 (d, *J* = 8.3 Hz, 2H), 7.51 (d, *J* = 8.3 Hz, 2H), 1.34 (s, 12H).^{13}C NMR (100 MHz, $CDCl_3$, ppm) δ 136.4 (CH), 131.0 (C), 131.0 (CH), 126.3 (C), 84.1 (C), 24.9 (CH_3). GC-MS (EI) *m/z* (relative intensity): 284 (56) [M^+], 282 (56), 269 (100), 267 (100), 198 (73), 196 (74), 185 (84), 186(95), 103(33).

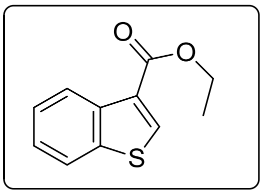

Ethyl benzo[*b*]thiophene-3-carboxylate: ^{1}H NMR (400 MHz, $CDCl_3$, ppm) δ 8.06 (s, 1H), 7.88 – 7.83 (m, 2H), 7.47 – 7.38 (m, 2H), 4.41 (q, *J* = 7.1 Hz, 2H), 1.42 (t, *J* = 7.1 Hz, 3H).^{13}C NMR (100 MHz, $CDCl_3$, ppm) δ 162.9 (CO), 142.2 (C), 138.8 (C), 133.9 (C), 130.4 (CH), 126.9 (CH), 125.5 (CH), 124.9 (CH), 122.8 (CH), 61.6 (CH_2), 14.4 (CH_3). GC-MS (EI) *m/z* (relative intensity): 206 (57) [M^+], 177 (50), 161 (100), 133 (24), 89 (40).

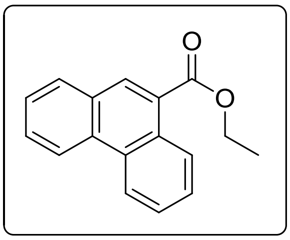

Ethyl phenanthrene-9-carboxylate: ^{1}H NMR (400 MHz, $CDCl_3$, ppm) δ 8.94 – 8.90 (m, 1H), 8.76 – 8.72 (m, 1H), 8.70 (d, *J* = 8.3 Hz, 1H), 8.47 (s, 1H), 7.98 (d, *J* = 8.0 Hz, 1H), 7.78 – 7.62 (m, 4H), 4.53 (q, *J* = 7.1 Hz, 2H), 1.51 (t, *J* = 7.1 Hz, 3H).^{13}C NMR (100 MHz, $CDCl_3$, ppm) δ 167.7 (CO), 132.1 (C), 132.1 (CH), 130.8 (C), 130.2 (C), 130.0 (CH), 129.1 (C), 128.9 (CH), 127.4 (CH), 127.0 (CH), 126.9 (CH), 126.7 (C), 126.7 (CH) 122.9 (CH), 122.7 (CH), 61.3 (CH_2), 14.5 (CH_3). GC-MS (EI) *m/z* (relative intensity): 250 (100) [M^+], 205 (87), 177 (71), 151 (16).

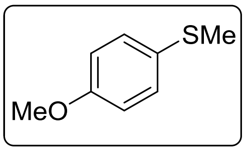

(4-Methoxy)thioanisole: ^{1}H NMR (400 MHz, $CDCl_3$, ppm) δ 7.28 (d, J = 8.8 Hz, 2H), 6.86 (d, J = 8.8 Hz, 2H), 3.79 (s, 3H), 2.45 (s, 3H). ^{13}C NMR (100 MHz, $CDCl_3$, ppm) δ 157.2 (C), 129.2 (CH), 127.8 (C), 113.6 (CH), 54.3 (CH_3), 17.1 (CH_3). GC-MS (EI) *m/z* (relative intensity): 154 (81) [M^+], 139 (100), 124 (7), 111 (18).

References

[1] Hari, D. P.; Schroll, P.; König, B. *J. Am. Chem. Soc.* **2012**, *134*, 2958-2961.

[2] Schroll, P.; Hari, D. P.; König, B. *ChemistryOpen* **2012**, *1*, 130-133.

[3] Hari, D. P.; Hering, T.; König, B. *Org. Lett.* **2012**, *14*, 5334-5337.

[4] Hering, T.; Hari, D. P.; König, B. *J. Org. Chem.* **2012**, *77*, 10347-10352.

[5] Xiao, T.; Dong, X.; Tang, Y.; Zhou, L. *Adv. Synth. Catal.* **2012**, *354*, 3195-3199.

[6] Majek, M.; Jacobi von Wangelin, A. *Chem. Commun.* **2013**, *49*, 5507-5509.

[7] Yu, J.; Zhang, L.; Yan, G. *Adv. Synth. Catal.* **2012**, *354*, 2625-2628.

[8] Szychlinski, J.; Bilski, P.; Martuszewski, K.; Blazejowski, J. *Analyst* **1989**, *114*, 739-741.

[9] Dakin, H. D. *Org. Synth.* **1935**, *15*, 74-75.

[10] Kuhn, H. J.; Braslavsky, S. E.; Schmidt, R. *Pure Appl. Chem*. **2004**, *76*, 2105-2146.

[11] Wegner, E. E.; Adamson, A. W. *J. Am Chem. Soc.* **1966**, *88*, 394-404.

[12] Mau, A. W.-H.; Johansen, O.; Sasse, H. F. *Photochem. Photobiol.* **1985**, *41*, 503-509.

[13] Lazarides, T.; McCormick, T.; Du, P.; Luo, G.; Lindley, B.; Eisenberg, R. *J. Am. Chem. Soc.* **2009**, *131*, 9192-9194.

Selected spectra:

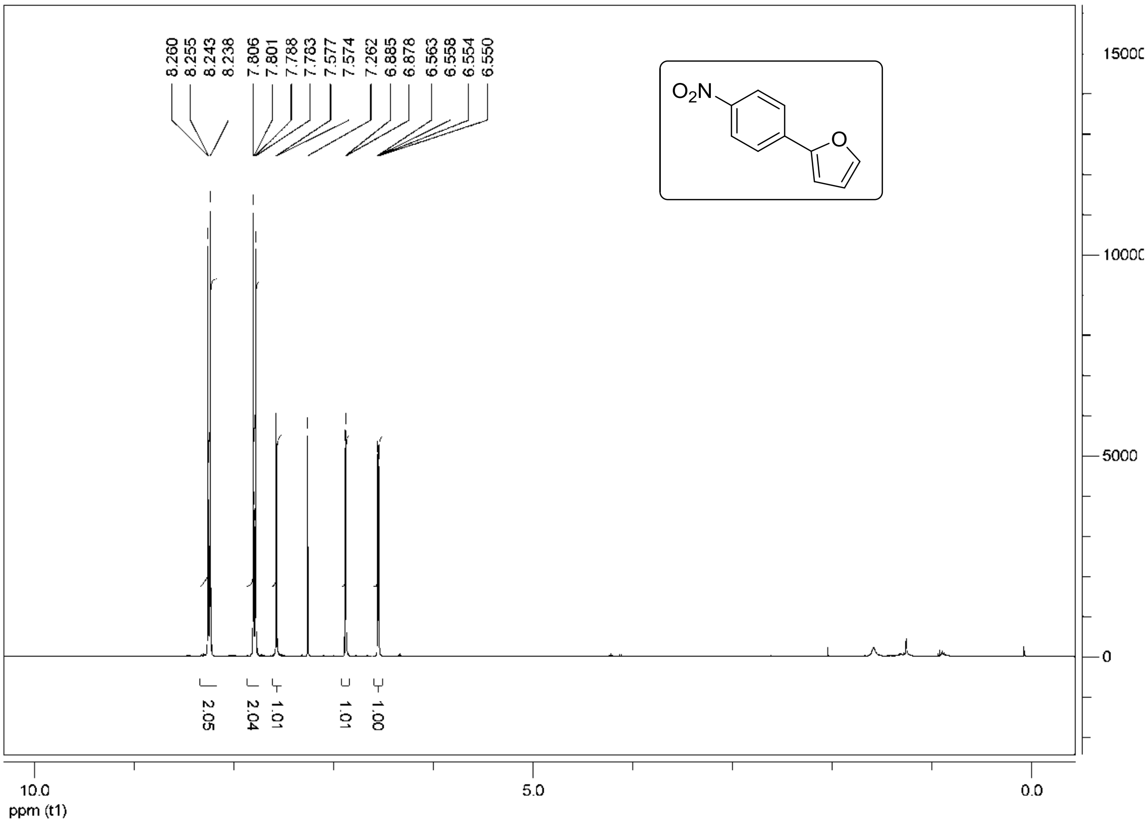

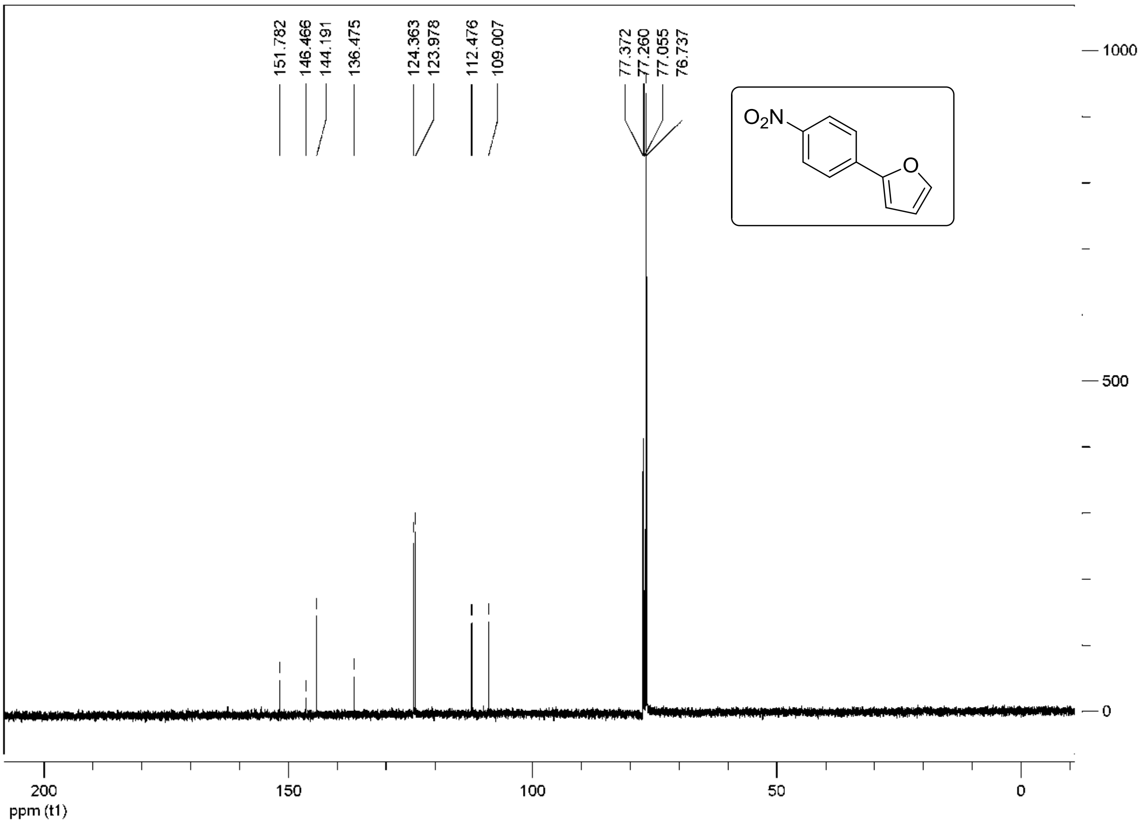

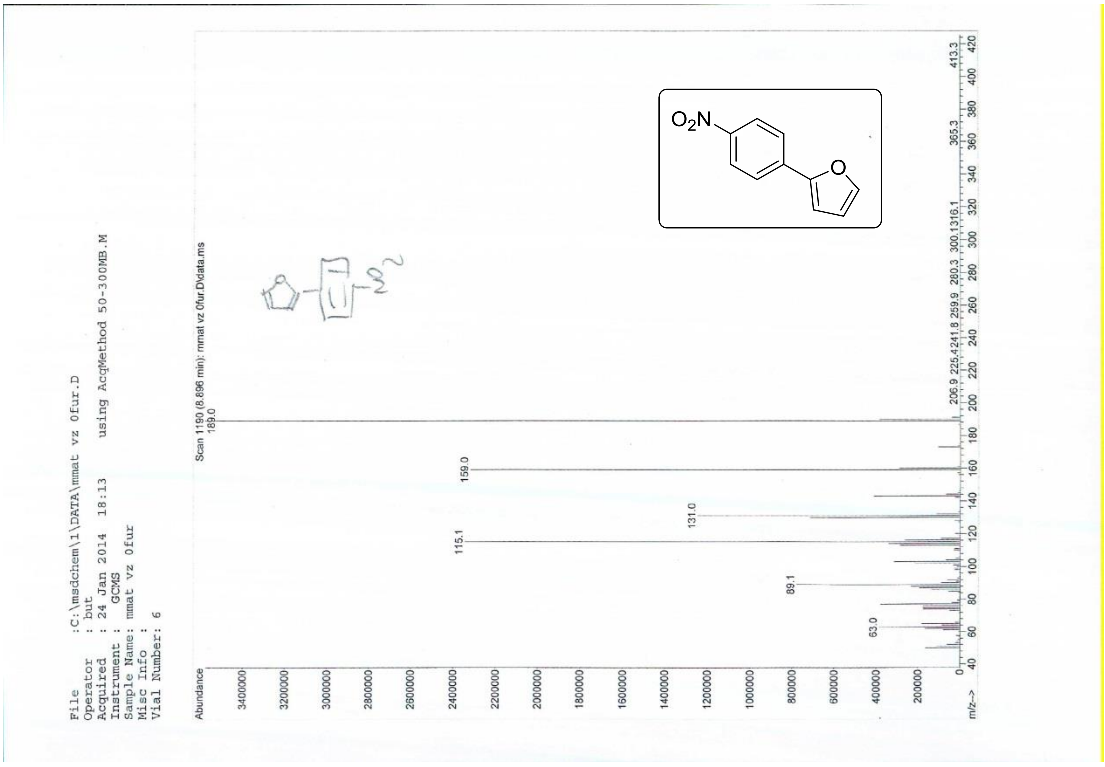

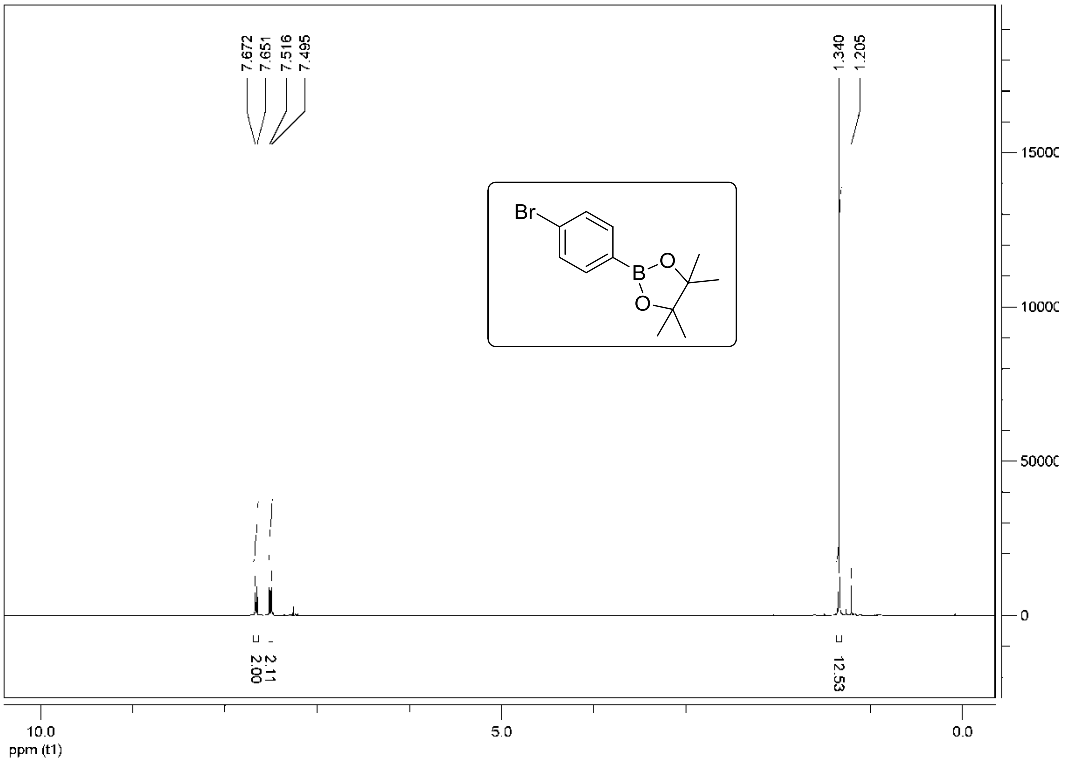
7.672
7.651
7.516
7.495
1.340
1.205
Br
B
O
O
15000
10000
50000
0
2.00
2.11
12.53
10.0
ppm (t1)
5.0
0.0

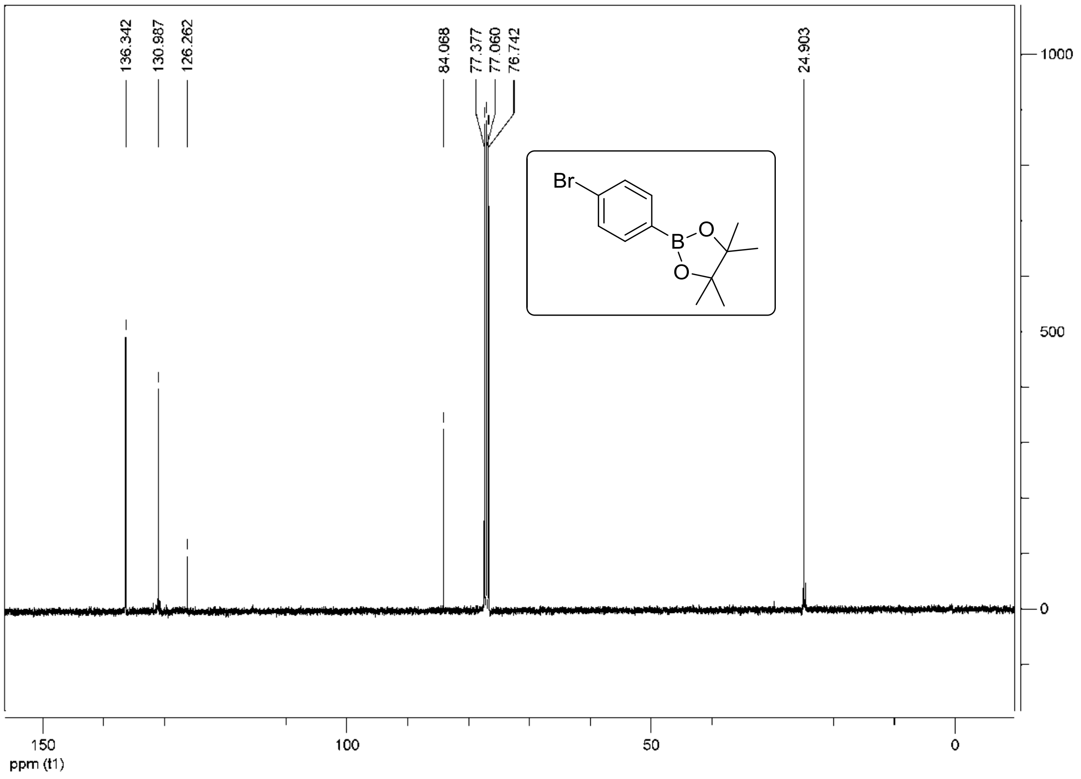
136.342
130.987
126.262
84.068
77.377
77.060
76.742
24.903
Br
B
O
O
1000
500
0
150
100
50
0
ppm (t1)

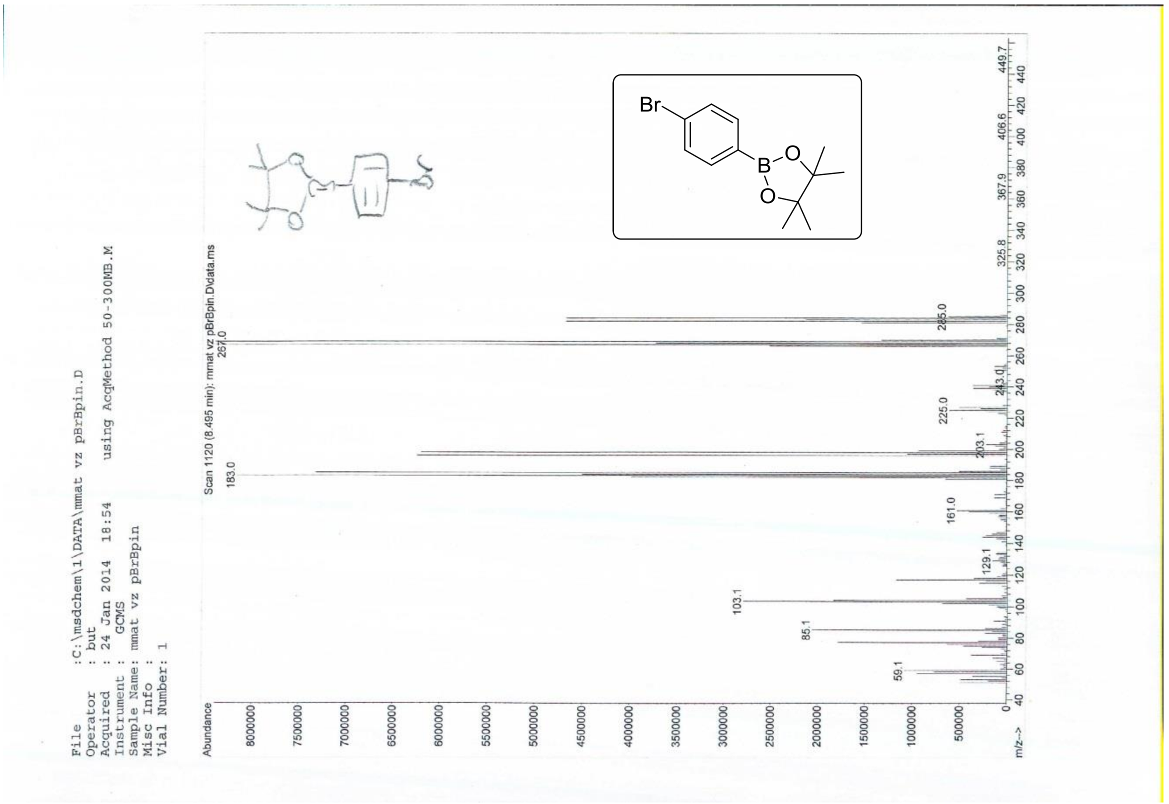
File :C:\msdchem\1\DATA\mmat vz pBrBpin.D
Operator : but
Acquired : 24 Jan 2014 18:54 using AcqMethod 50-300MB.M
Instrument : GCMS
Sample Name: mmat vz pBrBpin
Misc Info :
Vial Number: 1
Scan 1120 (8.495 min): mmat vz pBrBpin.D\data.ms
Abundance
m/z-->
183.0
267.0
285.0
225.0
243.0
203.1
161.0
129.1
103.1
85.1
59.1
325.8
367.9
406.6
449.7
Br
B
O
O

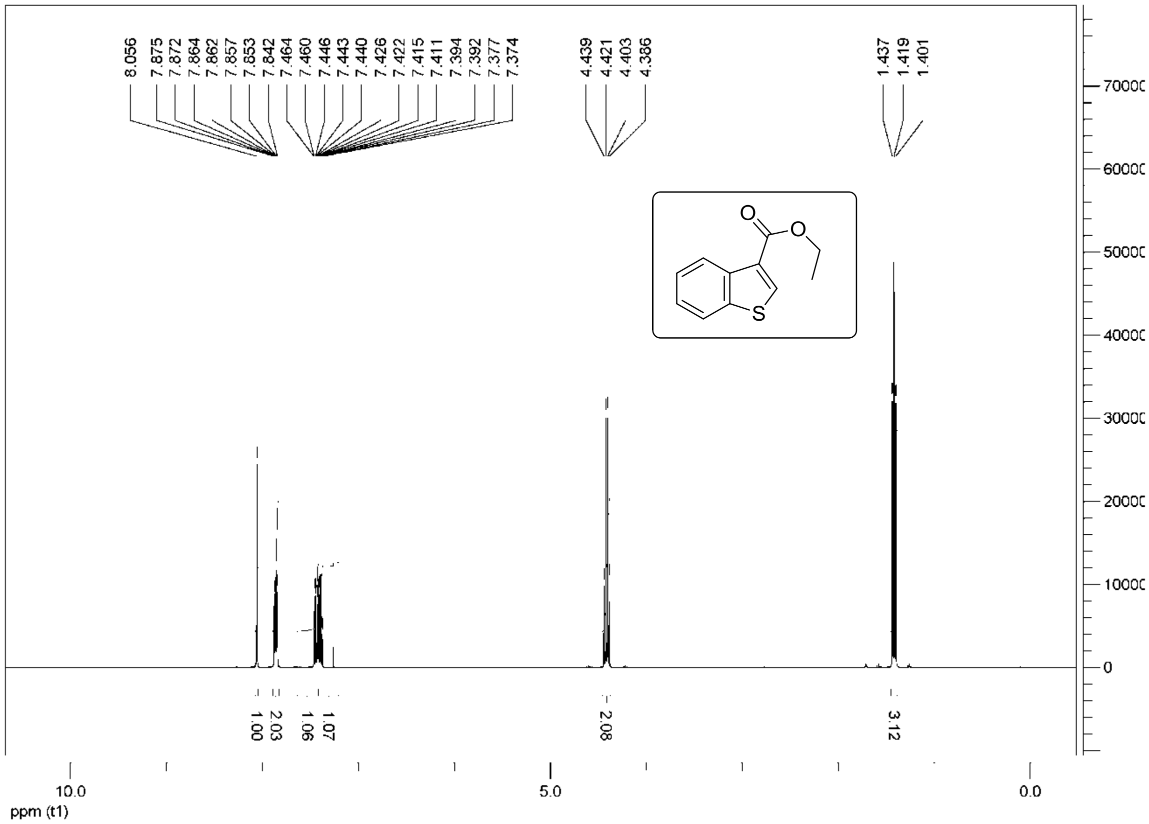
8.056
7.875
7.872
7.864
7.862
7.857
7.853
7.842
7.464
7.460
7.446
7.443
7.440
7.426
7.422
7.415
7.411
7.394
7.392
7.377
7.374
4.439
4.421
4.403
4.386
1.437
1.419
1.401
1.00
2.03
1.06
1.07
2.08
3.12
ppm (t1)
S

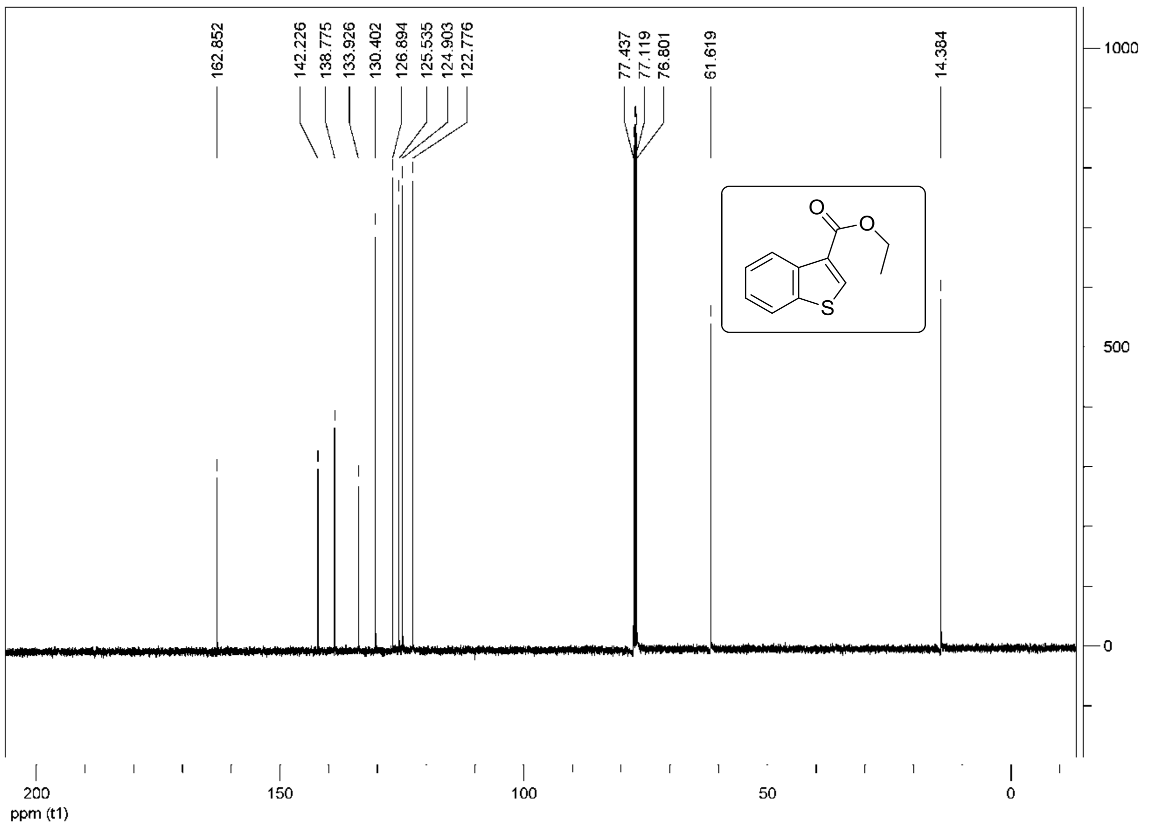
162.852
142.226
138.775
133.926
130.402
126.894
125.535
124.903
122.776
77.437
77.119
76.801
61.619
14.384
ppm (t1)
S

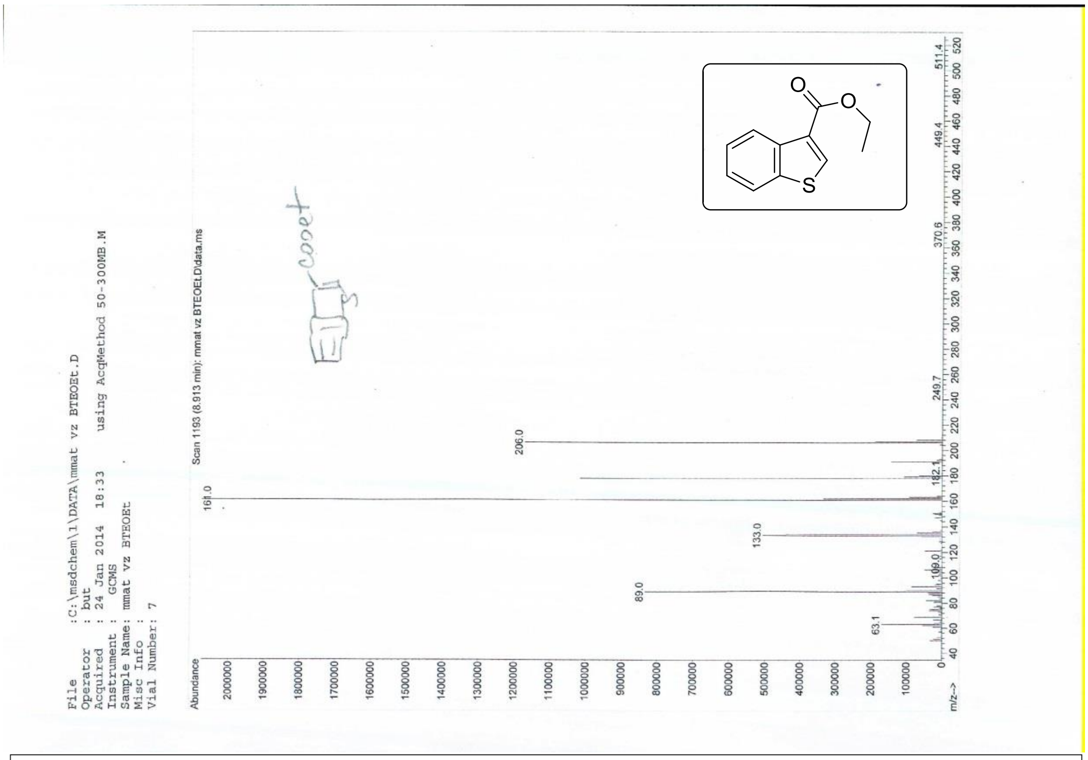
File :C:\msdchem\1\DATA\mmat vz BTEOEt.D
Operator : but
Acquired : 24 Jan 2014 18:33 using AcqMethod 50-300MB.M
Instrument : GCMS
Sample Name: mmat vz BTEOEt
Misc Info :
Vial Number: 7
Scan 1193 (8.913 min): mmat vz BTEOEt.D\data.ms
Abundance
m/z-->
161.0
206.0
182.1
133.0
89.0
63.1
249.7
370.6
449.4
511.4
O
O
S

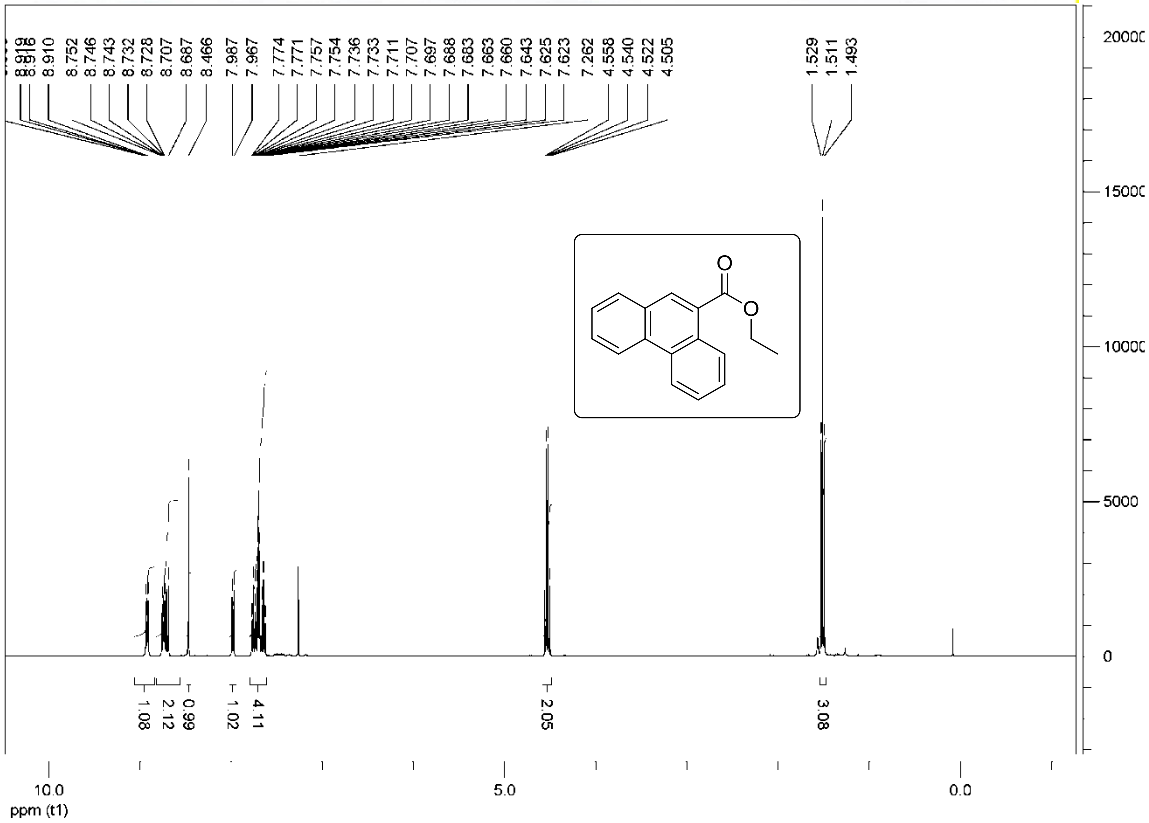
8.919
8.916
8.910
8.752
8.746
8.743
8.732
8.728
8.707
8.687
8.466
7.987
7.967
7.774
7.771
7.757
7.754
7.736
7.733
7.711
7.707
7.697
7.688
7.683
7.663
7.660
7.643
7.625
7.623
7.262
4.558
4.540
4.522
4.505
1.529
1.511
1.493
O
O
1.08
2.12
0.99
1.02
4.11
2.05
3.08
10.0
5.0
0.0
ppm (t1)
20000
15000
10000
5000
0

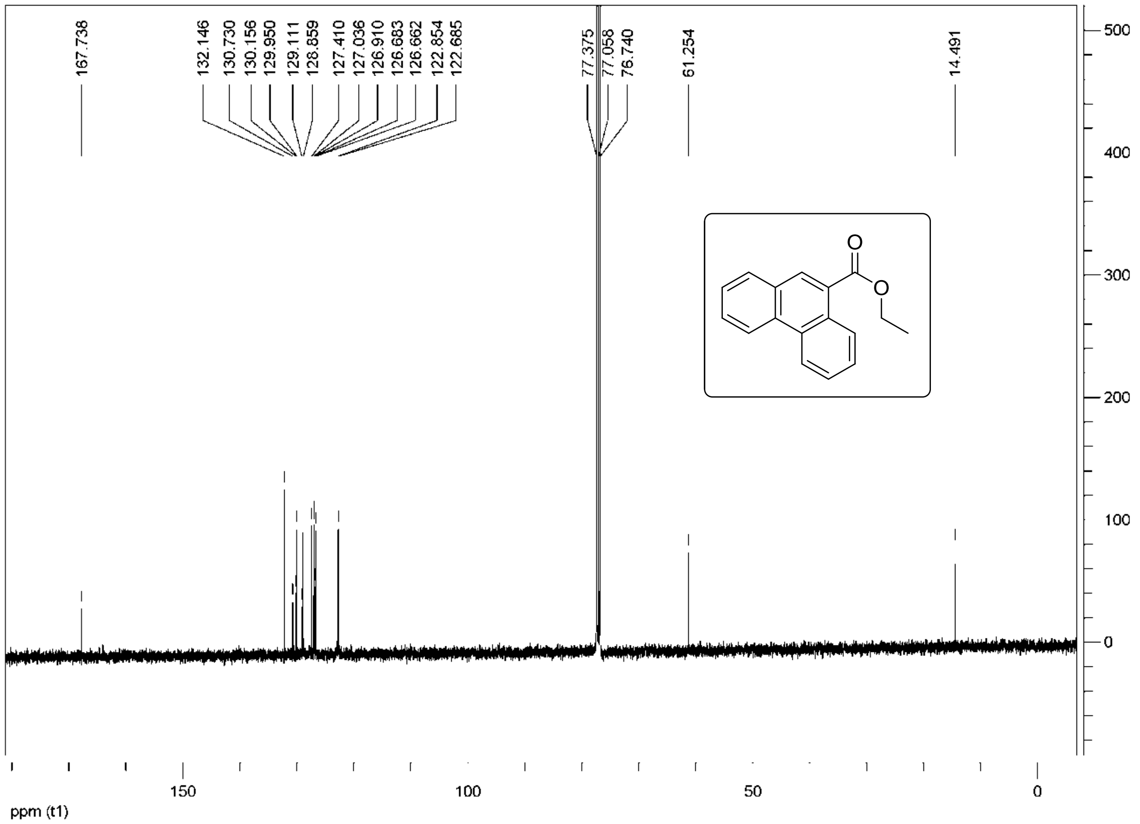
167.738
132.146
130.730
130.156
129.950
129.111
128.859
127.410
127.036
126.910
126.683
126.662
122.854
122.685
77.375
77.058
76.740
61.254
14.491
ppm (t1)

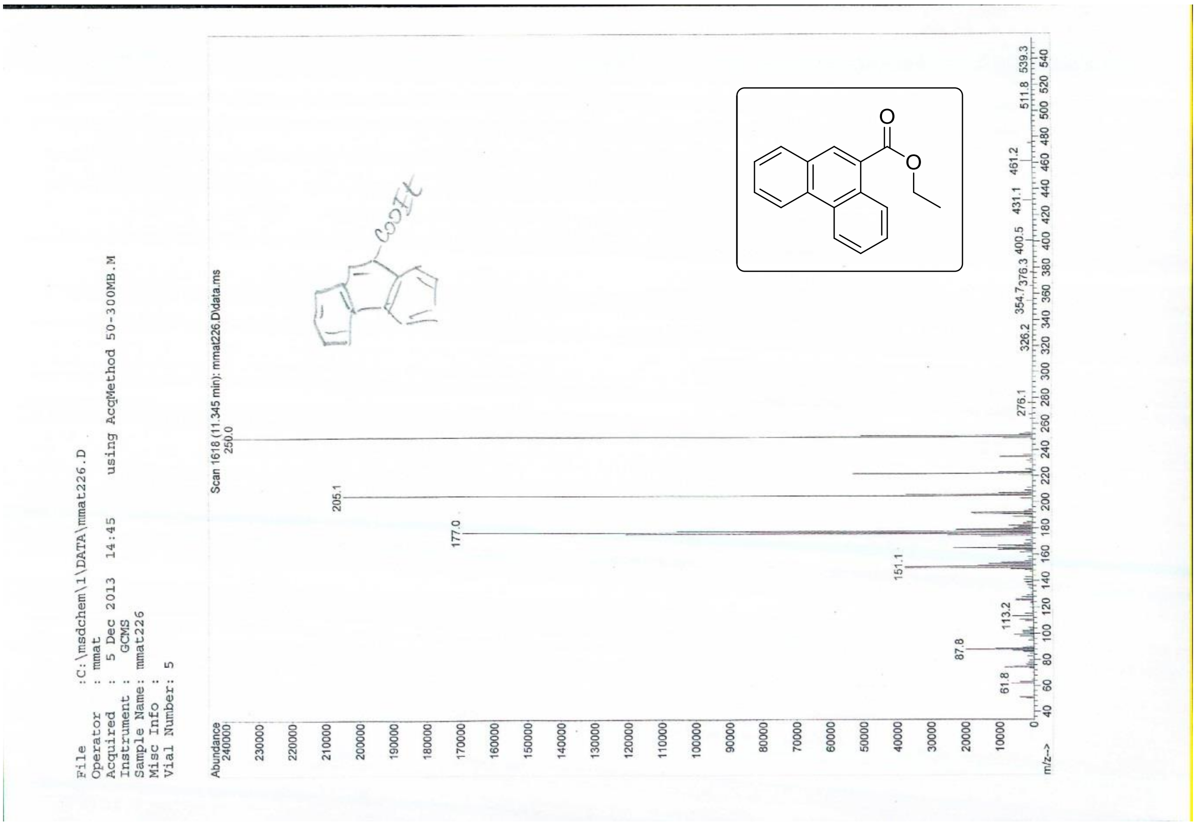
File :C:\msdchem\1\DATA\mmat226.D
Operator : mmat
Acquired : 5 Dec 2013 14:45 using AcqMethod 50-300MB.M
Instrument : GCMS
Sample Name: mmat226
Misc Info :
Vial Number: 5
Scan 1618 (11.345 min): mmat226.D\data.ms
Abundance
m/z-->

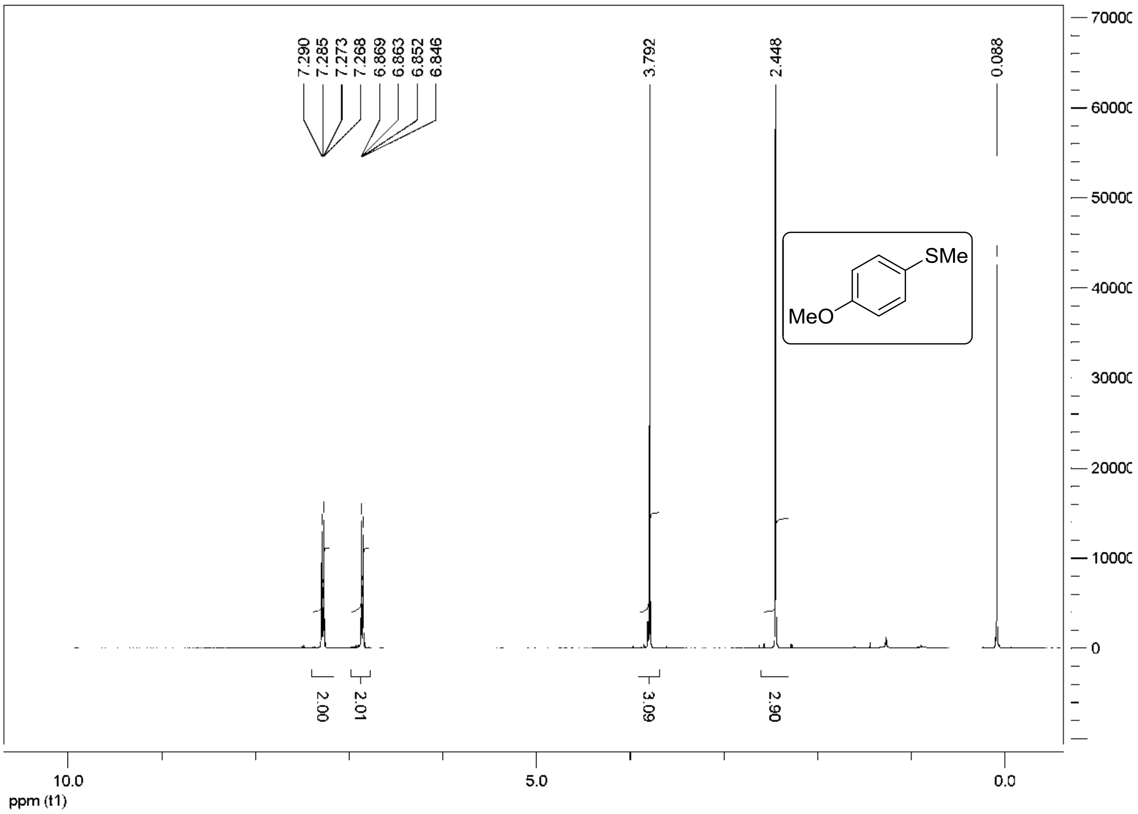
7.290
7.285
7.273
7.268
6.869
6.863
6.852
6.846
3.792
2.448
0.088
SMe
MeO
2.00
2.01
3.09
2.90
10.0
5.0
0.0
ppm (t1)

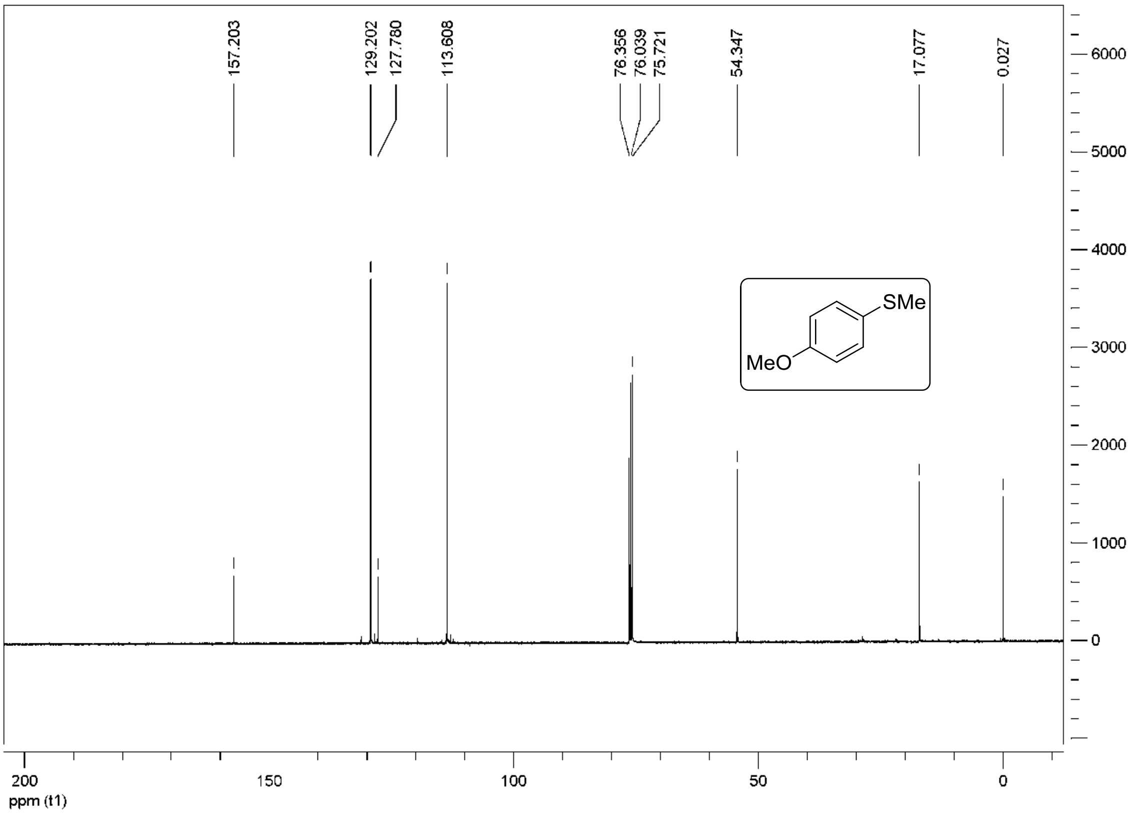
157.203
129.202
127.780
113.608
76.356
76.039
75.721
54.347
17.077
0.027
SMe
MeO
200
150
100
50
0
ppm (t1)

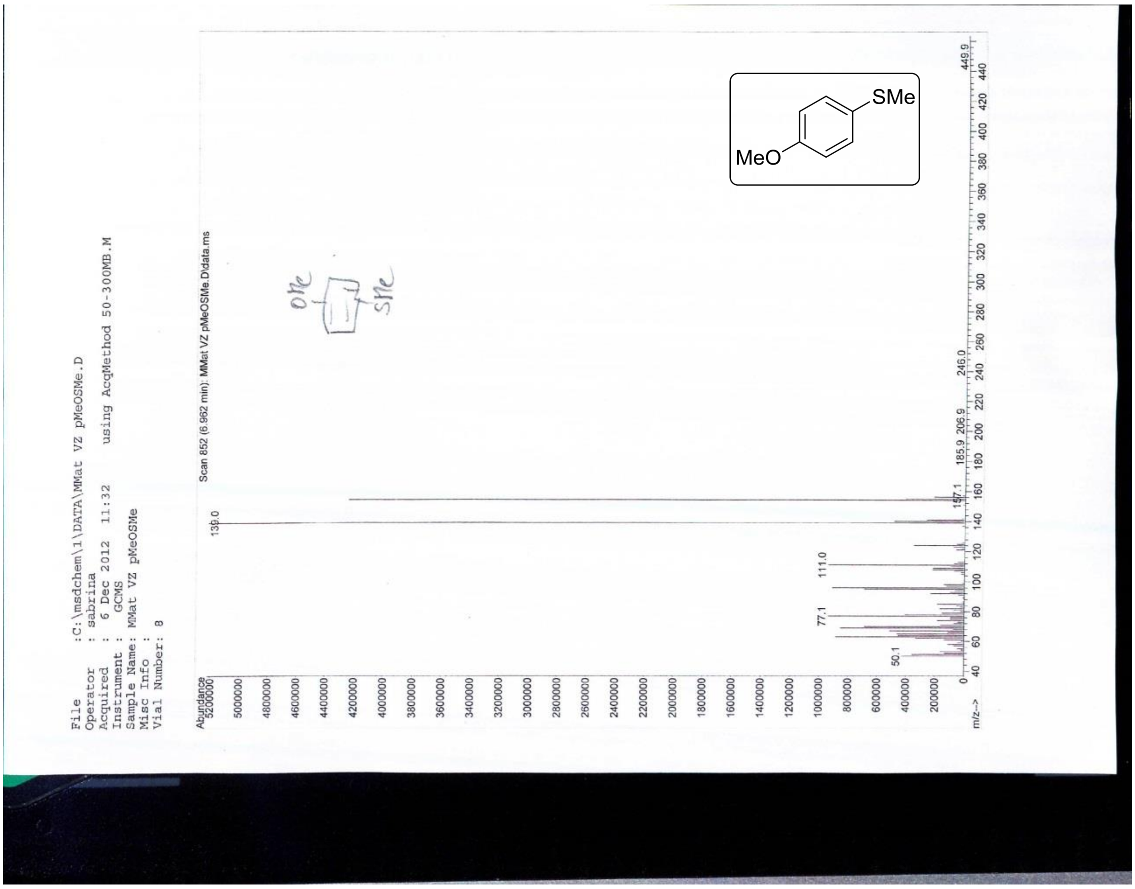
File :C:\msdchem\1\DATA\MMat VZ pMeOSMe.D
Operator : sabrina
Acquired : 6 Dec 2012 11:32 using AcqMethod 50-300MB.M
Instrument : GCMS
Sample Name: MMat VZ pMeOSMe
Misc Info :
Vial Number: 8
Scan 852 (6.962 min): MMat VZ pMeOSMe.D\data.ms
Abundance
m/z-->
139.0
111.0
77.1
50.1
157.1
185.9
206.9
246.0
449.9
SMe
MeO

Results of direct photolysis of *ortho*-biphenyldiazonium tetrafluoroborate in acetonitrile with broad spectrum CFL irradiation source.

```
File        :C:\msdchem\1\DATA\mmat228B.D
Operator    : mmat
Acquired    : 13 Dec 2013  10:35     using AcqMethod 50-300MB.M
Instrument  :    GCMS
Sample Name: mmat228B
Misc Info   :
Vial Number: 7
```

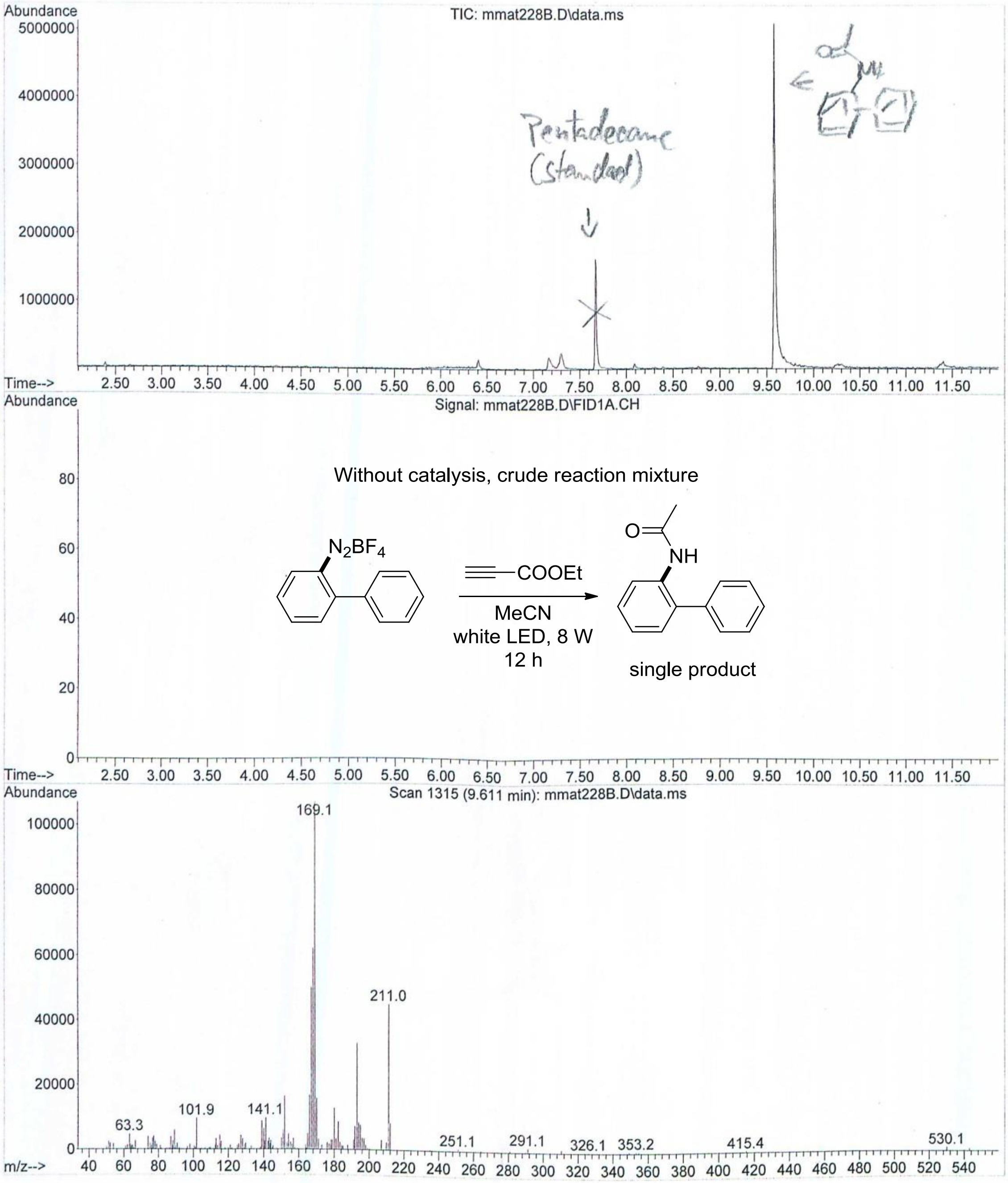

Supplement: File 1 — Experimental and analytical data. [file Beilstein_J_Org_Chem-10-981-s001.pdf]
